# HLA-A∗03:01 as predictive genetic biomarker for glatiramer acetate treatment response in multiple sclerosis: a retrospective cohort analysis

**DOI:** 10.1016/j.ebiom.2025.105873

**Published:** 2025-07-31

**Authors:** Brian C. Zhang, Tilman Schneider-Hohendorf, Rebecca Elyanow, Beatrice Pignolet, Simon Falk, Christian Wünsch, Marie Deffner, Erik Yusko, Damon May, Daniel Mattox, Eva Dawin, Lisa Ann Gerdes, Florence Bucciarelli, Lisa Revie, Gisela Antony, Sven Jarius, Christiane Seidel, Makbule Senel, Stefan Bittner, Felix Luessi, Joachim Havla, Matthias Knop, Manuel A. Friese, Susanne Rothacher, Anke Salmen, Fumie Hayashi, Roland Henry, Stacy Caillier, Adam Santaniello, University of California San Francisco MS-EPIC Team, Jessa Alexander, Riley Bove, Sergio Baranzini, Bruce A.C. Cree, Eduardo Caverzasi, Richard Cuneo, Stacy J. Caillier, Tiffany Cooper, Ari J. Green, Chu-Yueh Guo, Jeffrey M. Gelfand, Refujia Gomez, Sasha Gupta, Jill Hollenbach, Meagan Harms, Roland G. Henry, Stephen L. Hauser, Myra Mendoza, Jorge R. Oksenberg, Nico Papinutto, Sam Pleasure, Adam Santaniello, Joseph J. Sabatino, William A. Stern, Michael R. Wilson, Scott Zamvil, German Competence Network Multiple Sclerosis (KKNMS), Orhan Aktas, Antje Giede-Jeppe, Barbara Gisevius, Markus Kowarik, Friedemann Paul, Veit Rothhammer, Corinna Trebst, Uwe Zettl, Maria Seipelt, Christoph Heesen, Sandra Nischwitz, Antonios Bayas, Hayrettin Tumani, Florian Then Bergh, Gerd Meyer zu Hörste, Tania Kümpfel, Catharina C. Gross, Brigitte Wildemann, Martin Kerschensteiner, Ralf Gold, Sven G. Meuth, Frauke Zipp, Bruce A.C. Cree, Jorge Oksenberg, Michael R. Wilson, Stephen L. Hauser, Scott S. Zamvil, Luisa Klotz, Roland Liblau, Harlan Robins, Joseph J. Sabatino, Heinz Wiendl, Nicholas Schwab

**Affiliations:** a Adaptive Biotechnologies, Seattle, WA, USA; b Department of Neurology, University of Muenster, Muenster, Germany; c Toulouse Institute for Infectious and Inflammatory Diseases (Infinity), University of Toulouse, CNRS, INSERM, UPS, Toulouse, France; d Institute of Clinical Neuroimmunology, University Hospital, Ludwig Maximilian University Munich, Munich, Germany; e Biomedical Center, Faculty of Medicine, Ludwig Maximilian University Munich, Martinsried, Germany; f Munich Cluster of Systems Neurology (SyNergy), Munich, Germany; g Central Information Office German Competence Network of Multiple Sclerosis, Philipps University Marburg, Marburg, Germany; h Department of Neurology, University of Heidelberg, Heidelberg, Germany; i Department of Neurology, University of Leipzig, Leipzig, Germany; j Department of Neurology, University of Ulm, Ulm, Germany; k Department of Neurology, Focus Program Translational Neuroscience (FTN) and Immunotherapy (FZI), Rhine-Main Neuroscience Network (rmn), University Medical Center of the Johannes Gutenberg University Mainz, Mainz, Germany; l Max Planck Institute of Psychiatry, Munich, Germany; m Institute of Neuroimmunology and Multiple Sclerosis, University Medical Center Hamburg-Eppendorf, Hamburg, Germany; n Department of Neurology, University Hospital Hamburg-Eppendorf, Hamburg, Germany; o Department of Neurology and Clinical Neurophysiology, Medical Faculty, University of Augsburg, Augsburg, Germany; p Department of Neurology, St. Josef-hospital, Ruhr-University Bochum, Bochum, Germany; q Well Institute for Neurosciences, Department of Neurology, University of California, San Francisco, San Francisco, CA, USA; r Department of Neurology, University Hospital Marburg, Philipps-University Marburg, Marburg, Germany; s Department of Neurology, Medical Faculty, Heinrich-Heine University Düsseldorf, Düsseldorf, Germany; t Department of Neurology and Neurophysiology, University of Freiburg, Freiburg, Germany

**Keywords:** Glatiramer acetate, Multiple sclerosis, Biomarker, Treatment response, HLA, Genetics

## Abstract

**Background:**

Glatiramer acetate (GA) is a well-tolerated treatment for multiple sclerosis (MS) and comparable in its efficacy to high-dose interferon beta (IFN). As a lack of validated treatment response biomarkers for MS hampers progress in personalised treatment, the study goal was to search for biomarkers of a successful treatment response utilising the known observation of T-cell expansions after GA treatment.

**Methods:**

T-cell receptor beta chain (TRB) sequencing was performed in 3021 patients with MS: a discovery cohort of 1627 patients with MS, 204 of whom had previously been treated with GA, and then validated in 1394 patients with MS, 424 of whom had previously been treated with GA. Clinical data from 1987 patients with MS treated with GA or IFN and available HLA information from the NationMS, ACP, EPIC, BIONAT, and CombiRx trial cohorts were used for a subsequent analysis.

**Findings:**

Common GA-associated TRB expansions were exclusively detected in HLA-A∗03:01 or in HLA-DRB1∗15:01 backgrounds, within CD8+ effector- or CD4+ central-memory T cells. Both sets of common sequences clonally expanded after GA treatment in a first validation cohort and predicted GA exposure in two further validation cohorts. To evaluate whether restriction of public TRBs to only two HLA alleles is also associated with GA’s clinical efficacy, we analysed five cohorts of patients with MS for a potential benefit of the two HLAs concerning the GA response compared to IFN. We consistently found positive interactions with HLA-A∗03:01. This included a relative reduction in relapse risk compared to IFN in HLA-A∗03:01 carriers of 33% (CombiRx: GA + IFN arm: HR 0.67 [95% CI: 0.47–0.96], p = 0.0269) and 34% (CombiRx: GA arm: HR 0.66 [95% CI: 0.45–0.98], p = 0.0377), and in risk to first relapse of 63% (NationMS: HR 0.37 [95% CI: 0.16–0.88], p = 0.0246), but no positive association with DRB1∗15:01.

**Interpretation:**

HLA-A∗03:01 carrying patients with MS specifically benefit from GA treatment and GA significantly outperforms IFN in these patients. Therefore, determining HLA-A∗03:01 status before choosing a platform treatment for MS, would allow for a personalised treatment decision between GA and IFN.

**Funding:**

10.13039/501100001659German Research Foundation, 10.13039/100000002National Institutes of Health, 10.13039/100000890National Multiple Sclerosis Society, 10.13039/100020463Valhalla Foundation, Westridge Foundation, 10.13039/100026321Mayer Foundation, 10.13039/501100002347German Federal Ministry of Education and Research.

**Research in context**

**Evidence before this study**

We searched PubMed without language restriction until December 16th 2024 for relevant articles regarding glatiramer acetate (GA, also known as Copolymer-1 or Copaxone) and its clinical efficacy (also compared to interferon beta), its mechanism of action, and biomarkers for clinical response to GA treatment. The following search terms were used: “multiple sclerosis”, “glatiramer acetate”, “Copolymer-1”, “Copaxone”, “interferon beta”, “mechanism of action”, “biomarker”, “GWAS”, “clinical efficacy” and “clinical response”.

GA is an injectable peptide mix for the treatment of multiple sclerosis (MS), designed to mimic myelin basic protein composition. It has a superior safety profile, compared to other MS therapies, especially during pregnancy. Regarding its efficacy, it was shown to be comparable to high-dose interferon beta (IFN), which is the main alternative platform therapy. Since not all patients respond clinically to GA treatment, biomarkers have been sought to improve personalised patient care. Based on animal studies these screenings mainly focused on the HLA class II genetic background and associated immune cell types. Previous studies indicated a moderate beneficial effect for the MS risk allele HLA-DRB1∗15:01, or modulations of regulatory and helper T cells as well as B-cell reactions after GA treatment associated with a clinical response. Additional studies suggested HLA class I-restricted GA-specific T-cell expansions to be putatively associated with clinical benefit. However, none of the observed effects were deemed strong enough to be applicable in daily clinical practice.

**Added value of this study**

By performing next generation T-cell receptor sequencing from peripheral blood we could identify HLA-A∗03:01 allele carriers to respond to GA treatment with sequence-identical HLA class I-restricted T-cell expansions shared between patients, although all GA-treated patients with MS showed polyclonal expansions unique to each patient. A shared HLA class II-restricted response was also observed in patients carrying the MS risk allele HLA-DRB1∗15:01. When assessing clinical outcomes compared to IFN treatment in five large cohorts and -trials, HLA-A∗03:01 carriers specifically benefitted from GA treatment, while no independent effect could be observed for the HLA-DRB1∗15:01 MS risk allele. The previously observed moderate treatment response of HLA-DRB1∗15:01 carriers might be explained by the statistical overrepresentation of HLA-A∗03:01 in HLA-DRB1∗15:01 carriers due to their moderate linkage disequilibrium. GA treatment clearly outperformed IFN treatment in HLA-A∗03:01 carriers.

**Implications of all the available evidence**

Our study identifies HLA-A∗03:01 as a predictive and genetic treatment biomarker for MS, enabling caregivers and patients to make a personalised decision before initiation of treatment. Application of the biomarker offers one third of patients with MS (genetic GA responders) an efficacious treatment with a beneficial safety profile, superior to its comparator IFN and putatively within the range of escalation therapies. Secondly, the common GA-specific response in HLA-A∗03:01-carriers suggests a single antigenic peptide of the GA mixture to mediate its clinical effect, which offers the possibility to improve GA’s formulation and new insights to MS pathogenesis.

## Introduction

Multiple sclerosis (MS) is a chronic inflammatory and neurodegenerative disease affecting the central nervous system (CNS), where infiltrating, autoreactive T cells are thought to cause focal lesions by attacking the myelin sheath leading to demyelination, axonal loss, and glial scars.^1^ Initially designed to mimic myelin basic protein (MBP) amino acid composition, glatiramer acetate (GA) has become an established first-line treatment for relapsing-remitting MS^2^^,^^3^ and its prodrome, clinically isolated syndrome.^4^ Applied either daily or three times a week subcutaneously, GA reduces MS (relapse) activity to a similar degree as high-dose interferon beta (IFN).^5^^,^^6^ GA treatment can be viewed as a repeated, therapeutic vaccination, which elicits a variety of detectable immune responses. However, the exact mechanism of action (MOA) of GA is still unknown.^7^^,^^8^ While earlier theories of its MOA suggested GA being cross-reactive to MBP in a MHC-II-dependent manner,^9^ shifting the T-cell response from Th1 to Th2,^10^ inducing tolerance in the form of regulatory T cells^11^ or anti-inflammatory type II monocytes, the current understanding is that not all of these effects are antigen-dependent or even disease-specific.^12^ While it was observed that GA treatment can expand suppressor CD8+ T cells capable of killing CD4+ T cells in a GA-specific manner,^13^ not every induced effect of GA is necessarily associated with a clinical benefit. Recent studies have suggested that a clinically meaningful response to GA can be measured by assessing Th1/Th2 serum cytokine patterns^14^ or *in vitro* by detecting the presence of a B-cell reaction to brain lysate,^15^ but these measurements must be performed after some time on GA treatment.

There is currently no validated predictive treatment response biomarker for GA – or any other MS medication, for that matter.^16^ Patients with mild to moderate disease activity are usually started on a platform therapy, *i.e.,* IFN and GA.^5^^,^^6^ However, in a clinical setting, assessing tolerability and response to treatment for an individual patient can take months to years with the concomitant risk of disability accumulation.

The influence of the HLA locus on MS incidence has been firmly established. There are two primary HLA alleles associated with MS incidence in patients of European ancestry: HLA-DRB1∗15:01 increases MS incidence, whereas HLA-A∗02:01 confers a certain level of protection.^17^^,^^18^ However, HLA influence on MS severity is far less clear. Known MS risk single nucleotide polymorphisms within the HLA locus have been shown to influence age of disease onset, but no effect could be seen on disease burden after adjusting for the earlier onset, at least in progressive MS.^19^ HLA-A∗03:01 has been suggested as an additional MS risk allele,^20^^,^^21^ but larger studies have shown that this is mostly due to two indirect effects: i) more presence of HLA-A∗03:01 automatically results in less presence of HLA-A∗02:01 for a given haplotype, and ii) the linkage disequilibrium of HLA-A∗03:01 with HLA-DRB1∗15:01.^18^ As the view of GA as a therapeutic vaccination lends itself to the idea of a genetic, and especially an HLA, influence on its effects some studies have been performed to address this scientific question. These studies have so far focused on HLA class II genes and suggested a moderate effect of HLA-DRB1∗15:01 in the mediation of positive responses towards GA.^22^, ^23^, ^24^

The goal of this study was to leverage the known effect of GA on T-cell expansions, to i) identify specific T-cell receptor sequences responding to GA treatment resulting in better understanding of the MOA of GA, ii) uncover the genetic determinants underlying common T-cell expansions, and iii) use this to delineate genetic predictors to stratify patients prior to starting IFN or GA therapy in the form of personalised medicine.

## Methods

### Patient cohorts

For this study we analysed several cohorts of patients with MS. Some cohorts were analysed regarding their T-cell receptor beta chain (TRB) repertoire, some of them clinically, and some of them regarding both modalities (Table 1): The “Accelerated Cure Project” cohort (ACP) is derived from a repository of blood-derived samples collected from more than 3200 participants at 10 major MS centres located across the United States under a standardised protocol. The “BIONAT” cohort was collected by a French study registry and consists of patients with MS before and during natalizumab treatment, some of them with prior GA treatment.^25^ The “CombiRx” cohort is derived from the CombiRx trial, which was a 3-arm, randomised, double-blind, placebo-controlled, multi-centre, Phase-III trial comparing IFN to GA or to IFN + GA in patients with RRMS. The “Expression, Proteomics, Imaging, Clinical” (EPIC) cohort was collected by the University of California, San Francisco and comprises patients with MS from the United States with roughly 80% European ancestry.^26^ The “Finnish Biobank Cooperative” (FinBB) cohort contains blood-derived samples from six Finnish biobanks: Auria Biobank, Helsinki Biobank, Biobank of Eastern Finland, Central Finland Biobank, Borealis Biobank, and Tampere Biobank. The “MS twin” cohort is collected at the Institute of Clinical Neuroimmunology at the Ludwig Maximilian University of Munich, Germany, and consists of patients with MS and their healthy, syngeneic twin sibling. The “Muenster” cohort is a monocentric collection of early patients with MS, mostly untreated. The “NationMS” cohort is collected by the Disease-Related Competence Network of Multiple Sclerosis (KKNMS, Germany) and consists of initially treatment-naive patients with CIS and RRMS according to the 2005 revisions to the McDonald diagnostic criteria for MS, followed over a period of at least ten years. For all presented cohorts, sex was self-reported by participants (Table 1).

**Table 1:** Patient cohort characteristics.

Cohort	Time structure	Time points	Parameters (patient n)	Diagnosis	Age [years]	Sex ratio [f:m]	Disease duration [years]
ACP	Longitudinal	Twice during therapy with GA or IFN	TRB (309)/Clinical (148)	91.1% RRMS, 7.4% SPMS, 1.5% PPMS	43.4	3.9:1	8.9
BIONAT	Cross-sectional	Once after prior therapy with GA or no therapy with GA	TRB (1255)/Clinical (600)	97.2% RRMS, 1.8% SPMS, 0.7% inconclusive, 0.3% PPMS	36.8	4.6:1	8.7
CombiRx	Longitudinal	Once before therapy with GA, IFN or both and up to 21 times during therapy	Clinical (693)	100% RRMS	37.7	2.6:1	4.3
EPIC	Longitudinal	Once before and once during therapy with GA or IFN	TRB (72)/Clinical (125)	83.9% RRMS, 6.1% SPMS, 6.7% CIS, 2.8% PPMS, 0.5% inconclusive	41.1	3.5:1	6.8
FinBB	Cross-sectional	Once during therapy with GA or no therapy with GA	TRB (1049)	98.6% MS, 1.4% suspected CIS	49.2	3.1:1	8.6
MS twin	Cross-sectional	Once during therapy with GA or no therapy with GA	TRB (67)	71.6% RRMS, 17.9% SPMS, 6% CIS, 3% PPMS, 1.5% inconclusive	40.8	3.2:1	11.7
Muenster	Cross-sectional	Once after prior therapy with GA or no prior therapy with GA	TRB (94)	84% RRMS, 9.6% CIS, 5.3% PPMS, 1% SPMS	37.6	2.2:1	4.3
NationMS	Longitudinal	Once before therapy with GA or IFN and, one year apart, once during therapy	TRB (246)/Clinical (342)	54.7% RRMS, 45.3% CIS	33.9	2.7:1	0.55

RRMS = relapsing-remitting multiple sclerosis, SPMS = secondary progressive multiple sclerosis, PPMS = primary progressive multiple sclerosis, CIS = clinically isolated syndrome, TRB = T-cell receptor beta chain, GA = glatiramer acetate, IFN = interferon beta; Age and disease duration are given as means.

### Ethics

The study was conducted in accordance with the Declaration of Helsinki. The NationMS study was approved by the ethics committee of the Ruhr-University Bochum (registration no. 3714–10) and, consecutively, by all local committees of the participating centres. Written informed consent was obtained from all study participants.

### Role of funders

The funders did not have any role in study design, data collection, data analyses, interpretation, or writing of report.

### Bulk T-cell receptor beta chain sequencing

Genomic DNA was extracted from frozen, plasma-depleted blood samples or PBMC using the Qiagen DNeasy Blood Extraction Kit (Qiagen) either in Muenster, Toulouse, or in Seattle. As much as 18 μg of input DNA was then used to perform immunosequencing of the third complementarity determining (CDR3) regions of the TRB using the ImmunoSEQ Assay (Adaptive Biotechnologies). Briefly, input DNA was amplified in a bias-controlled multiplex PCR, followed by high-throughput sequencing. Sequences were collapsed and filtered to identify and quantitate the absolute abundance (*i.e.,* templates) of each unique TRB CDR3 region for further analysis, as previously described.^27^ An expansion was defined as a nucleotide-level rearrangement not present in the baseline sample, but being in the top 100 most prevalent rearrangements in the sample after treatment. Productive clonality was calculated as described previously.^28^ For the current study, we integrated Immunoseq samples from 20 batches, which were sequenced over the course of 7 years. While variation in flowcell density can result in some difference in coverage, the data remains replicable and the minimum quality standards ensure that all passing samples are sequenced to sufficient coverage such that nearly all unique species in a sample are seen and quantified. PCR amplification bias and coverage are all measured and adjusted based on in-line controls (*i.e.,* primers to reference genes that enable accurate post-sequencing quantitation of total nucleated cells in each sample as QC). In addition, the synthetic immune repertoire, which is included in every sample and sequencing run, serves as a positive in-line control for PCR and sequencing performance, enabling to quantitate individual TRB sequences from sequencing read counts.^29^ The assay design allows to address sequencing and PCR errors. Differences in sample quality, extraction efficiency, or cell sorting may affect the total amount of data but reported results are all standardised with minimal batch effects.

### Single-cell RNA sequencing and analysis

PBMC were collected from EDTA blood and then diluted in X-Vivo15 medium (Lonza Bioscience), sorted for CD3+ T cells (Miltenyi Biotec) and loaded onto the Chromium Single Cell Controller using the Chromium Next GEM Single Cell 5’ Kit v2 (10× Genomics) chemistry for a maximum input of 10,000 cells, following the manufacturer’s instructions. Sample processing and library preparation for transcriptome and T-cell receptor repertoire was performed according to manufacturer instructions using AMPure beads (Beckman Coulter). Sequencing was carried out on a local Illumina NovaSeq 6000 using the XP reagent kit with a 26-10-10-90 read setup. Processing of sequencing data was performed with the cellranger pipeline v7.1.0 (10× Genomics). Raw bcl files were demultiplexed using cellranger mkfastq. Subsequent read alignment and transcript counting were done using cellranger multi for each sample individually. Cellranger output files were analysed using Seurat workflow and Azimuth,^30^ as well as aCSF^31^ annotation. For matching of TRBs between bulk and single-cell sequencing a Hamming distance of 1 was allowed.

### Statistics

#### Patient matching

Due to the cross-sectional nature of the BIONAT cohort, 839 patients were matched for the parameters age, sex, HLA-A∗03:01 and HLA-DRB1∗15:01 between groups with and without prior treatment with GA. The matching was performed using the R package MatchIt^32^ with the method “exact” and the distance “glm” resulting in two matched groups of 367 patients with and 235 patients without prior treatment with GA.

#### Discovery of GA-associated TRBs

In each of the three cohorts in the training set, we separately assessed the frequency of each TRB in samples of patients with MS with positive (“cases”) or negative (“controls”) GA exposure status. The training set included 58 GA-treated cases and 211 controls from the combined NationMS and Muenster cohort, 75 cases and 234 controls from the ACP cohort, and 71 cases and 978 controls from the FinBB cohort. Statistical enrichment was determined using a one-sided Fisher’s Exact Test (FET). TRBs with a p-value below a threshold of 0.001 were counted as Enhanced Sequences (ES). To allow for discovery of private TRBs sharing a common, public motif, we further expanded our ES by computing FET p-values for all “one-wildcard” matches to ES (*e.g.,* for TRB CASSSHGGEQYF + TRBV05-06 + TRBJ02-07, a one-wildcard motif might be CASSxHGGEQYF + TRBV05-06 + TRBJ02-07, where x could be any amino acid). We selected as ES one-wildcard motifs where the combined p-value was less than 0.0001 and less than the p-value of any individual TRB in the motif. Lastly, to control for cohort effects, we required ES to be significantly enriched in at least two cohorts in the training set. This resulted in a final set of 105 GA-associated ES. We chose to not apply false discovery rate (FDR) correction in the ES discovery step because statistical stringency was achieved through a dual-filter strategy: a low p-value threshold (p < 0.001) combined with multi-cohort replication. This approach has been shown, *e.g.,* in GWAS studies, to minimise false positives by requiring consistency across cohorts.^33^ Furthermore, using replication across cohorts can act as an empirical filter and be more robust than theoretical multiple testing corrections when datasets are heterogeneous.^34^

### Determination of HLA association of GA-associated TRBs

We mapped these 105 TRBs to HLAs based on statistical association. First, HLA classifiers trained as described previously^35^ were used to label each of the individuals in the study as positive or negative for each of 145 modelled HLAs. Briefly, these models predict the presence or absence of an HLA allele using logistic regression on observed HLA-associated TRB sequences and log total unique rearrangement counts, as described previously.^36^ They were trained via 5-fold cross validation on 4144 individuals with HLAs genotyped via next generation sequencing and found to have AUROC > 0.9 on a holdout dataset. Once each individual was assigned HLA labels, each of the 105 GA-associated TRB sequences were tested against each HLA label using one-sided Fisher's Exact Tests. We then selected the most significant predicted HLA, or in cases of a common motif, the most significant HLA among TRBs with the motif. This resulted in 65 HLA-A∗03:01-associated TRBs, 35 HLA-DRB1∗15:01-associated TRBs, and 5 TRBs with uncertain or other HLA: three TRBs mapped to other HLAs (C∗02:02, DRB1∗01:01, DRB5∗02:02) at a p < 0.0001, as well as two uncertain TRBs A∗03:01 and DRB1∗04:01 at a suggestive p < 0.001. The HLA-C∗02:02 TRB association was identified by leveraging typed HLA samples, as this was not one of the 145 imputed HLAs.

Given a TRB repertoire, we computed its HLA-A∗03:01 GA clonal breadth as the number of unique rearrangements matching one of the 65 A∗03:01-associated TRBs as a fraction of the total unique productive rearrangements. We computed its HLA-A∗03:01 GA clonal depth as the number of templates matching the 65 A∗03:01-associated TRBs as a fraction of the total productive templates. HLA-DRB1∗15:01 GA clonal breadth and depth were defined analogously.

### Clinical parameters and modelling

For the CombiRx analyses, we obtained data used in the 7-year CombiRx follow-up,^37^ including HLA typing data for 700 of the 1008 patients. For HLA class I only two-digit information was available. Before introducing HLA, we were able to exactly replicate measures for annualised relapse rate (sum of protocol defined, non-protocol defined, and suspected exacerbations), combined unique activity (CUA), and percent change lesion volume as reported.^37^ Annualised relapse rate was computed over the entire follow-up period, while for the longitudinal measures we analysed changes per year at the three-year mark. Note that CombiRx used 28-day months to determine visits while in plots we used “years” to indicate a period of 12 28-day months. For the EPIC, NationMS, and BIONAT cohorts, we analysed clinical outcomes alongside molecular HLA typing. For the ACP cohort, we used HLA imputation^35^ to infer HLA-A∗03:01 and HLA-DRB1∗15:01 status from immunosequencing of blood. Depending on the analysed variable, we applied either a linear model (delta EDSS, MSSS, delta MSFC, delta Nfl, delta lesion number, delta CUA, percent change lesion volume), a linear mixed model with the subject as random intercept (number of Gd-enhancing lesions), a Cox regression (time to first relapse), or a Cox regression with Andersen-Gill modification (all relapses). The covariates for each model are shown in the respective plots and homogeneously included age, sex, disease duration, treatment, HLA-A∗03(:01), HLA-DRB1∗15:01 and the interactions between treatment and HLAs. Age and disease duration are normalised by min max scaling to improve visualisation within plots without influencing effect sizes or significances of other covariates.

### Role of funders

The funders had no role in study design, data collection, data analyses, interpretation, or writing of report.

## Results

### Treatment with glatiramer acetate expands HLA-A∗03:01-associated CD8+ T cells and HLA-DRB1∗15:01-associated CD4+ T cells with shared TRB sequences and motifs

**Fig. 1:**
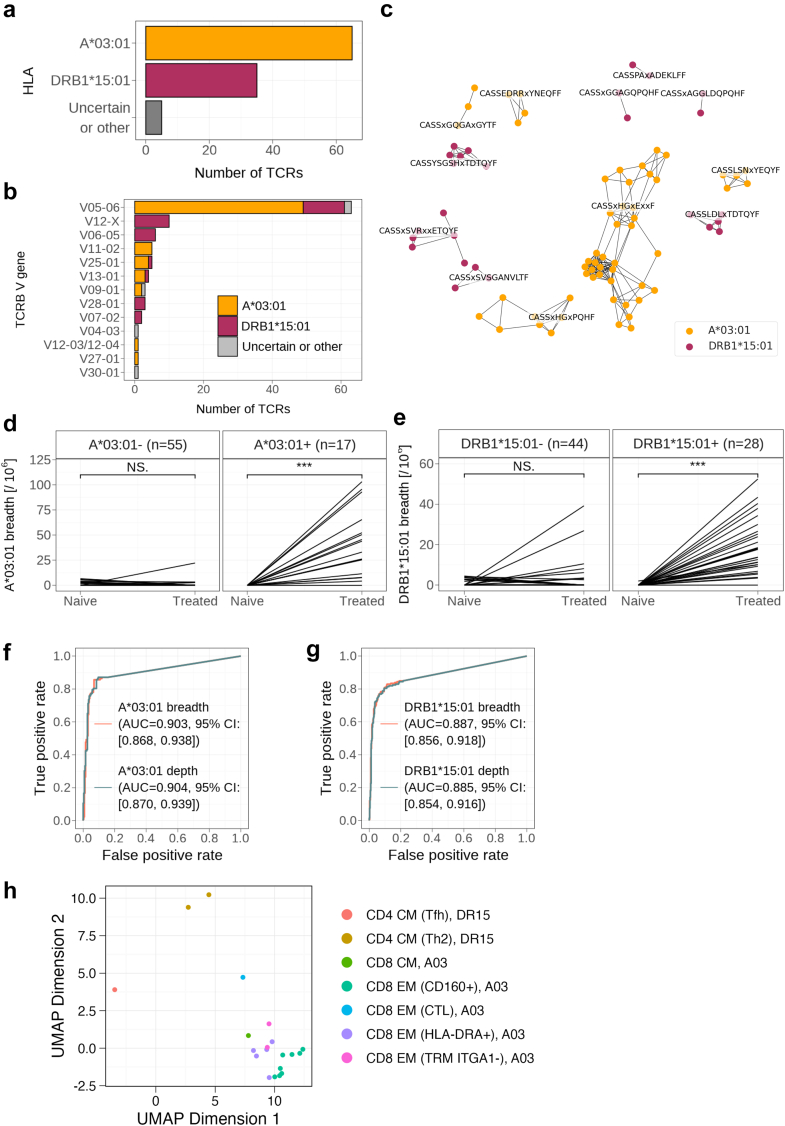
**Treatment with glatiramer acetate expands HLA-A∗03:01-associated CD8+ T cells and HLA-DRB1∗15:01-associated CD4+ T cells with shared TRB sequences and motifs.** a: Number and HLA restriction of common GA-associated TRB sequences identified from bulk TRB sequencing of GA-treated *vs.* non-GA-treated patients with MS. b: Variable beta chain gene usage of GA-associated TRB sequences. c: 1-Hamming clustering of GA-associated TRB sequences with resulting highlighted motifs. d and e: Validation of HLA-A∗03:01-associated (d, n(HLA-A∗03:01 negative) = 55, p = 0.82, n(HLA-A∗03:01 positive) = 17, p = 0.00011) and HLA-DRB1∗15:01-associated (e, n(HLA-DRB1∗15:01 negative) = 44, p = 0.16, n(HLA-DRB1∗15:01 positive) = 28, p < 0.0001) GA-associated TRB sequence patterns in TRB repertoires before (Naive) and after treatment (Treated) with GA in the EPIC cohort by quantification of GA-associated TRB clonal breadth per 10^6^ unique rearrangements. Significance was assessed by paired t-test. f and g: AUC curves predicting history of GA treatment in HLA-A∗03:01 positive patients (f) and HLA-DRB1∗15:01 positive patients (g) from validation cohorts (BIONAT (n = 1255), MS twin (n = 67)). h: Uniform manifold approximation and projection (UMAP) plot showing CD3+ sorted T cells expressing GA-associated TRB sequences from three HLA-A∗03:01 positive and HLA-DRB1∗15:01 positive GA-treated patients with MS, annotated by Seurat and aCSF clustering (aCSF annotations in brackets, TRB sequence HLA association in square brackets). As shorthand, A03 and DR15 are used to refer to HLA-A∗03:01 and HLA-DRB1∗15:01. Significance was assessed by a paired Wilcoxon rank-sum test. The asterisks indicate a p-value of <0.001(∗∗∗).

Bulk TRB sequencing was performed on 1627 patients with MS (FinBB: 1049, ACP: 309, NationMS and Muenster: 269). While patients in general showed clonal expansions after GA treatment (Supplementary Fig. S1a), analysis by comparing patients with GA exposure (n = 204) to a large amount of patients without exposure to GA (n = 1423) revealed two patterns of common, GA-associated sequences. 65 sequences defined an HLA-A∗03:01-associated pattern and 35 sequences defined an HLA-DRB1∗15:01-associated pattern; 5 additional sequences were significantly associated with GA, but with no other HLA appearing multiple times (Supplementary Table S1; Fig. 1a).

The variable beta chain distribution of these patterns was strongly skewed towards TRBV5-6 with 63 of 105 sequences (60%) expressing this gene (Fig. 1b). Hamming distance clustering of the two patterns revealed strong similarities between the sequences, with five clusters in the HLA-A∗03:01 pattern and seven clusters in the DRB1∗15:01 pattern. The HLA-A∗03:01 pattern revealed a strong TRBV5-6 motif CASSxHGxxxxF in 44 of 65 sequences (Fig. 1c).

The A∗03:01 and DRB1∗15:01 sequence patterns were validated in a separate cohort of 72 patients with MS before and after GA treatment (EPIC: n = 72) and both were significantly expanded in their respective HLA backgrounds (Fig. 1d and e). The patterns also accurately predicted prior GA exposure (AUC: HLA-A∗03:01: 0.90 [95% CI: 0.87–0.94], DRB1∗15:01: 0.89 [95% CI: 0.86–0.92]) in two additional cohorts of 1322 individuals (BIONAT: n = 1255, MS twin: n = 67; Fig. 1f and g). Of note, pattern clonal breadth (number of unique TRB rearrangements in a sample fitting the pattern) and clonal depth (number of TRB templates/cells in a sample fitting the pattern) performed similarly well. After cessation of GA therapy, both breadth and depth of the two patterns decreased with time (Supplementary Fig. S1b–e). Interestingly, there was a genetic synergism in which patients who were HLA-DRB1∗15:01 positive in addition to being HLA-A∗03:01 positive presented with more HLA-A∗03:01 pattern-matched TRB rearrangements compared to patients who were only HLA-A∗03:01 positive (Supplementary Fig. S1f and g). This was not observed for the HLA-DRB1∗15:01 pattern (Supplementary Fig. S1h and i).

Further characterisation was performed using single-cell RNA sequencing of sorted T cells from a fourth validation cohort of GA-treated patients with MS who were HLA-A∗03:01 and HLA-DRB1∗15:01 positive. Analysis showed that the HLA-DRB1∗15:01 sequence-matched cells were CD4+ central-memory T cells with a T-helper 2/T-follicular helper transcriptome, and the HLA-A∗03:01 sequence-matched cells were CD8+ T cells exhibiting different effector-memory transcriptomes, mostly CD160+, or HLA-DR+, or ITGA1-tissue-resident memory cells^31^ (Supplementary Table S2; Fig. 1h). The T-cell receptor alpha chains of these matched cells also showed high similarities. Of note, to characterise the quality of these expansions in their respective HLA backgrounds, TRB sequencing of a fifth validation cohort of sorted CD8+ and CD4+ T cells before and after GA treatment^38^ was analysed. A more focused immune response was observed in CD8+ T cells of HLA-A∗03:01 carrying patients with lower clonality after one year of GA treatment compared to HLA-A∗03:01 non-carrying patients, while no differences could be detected for CD4 T-cell expansions and HLA-DRB1∗15:01 (Supplementary Fig. S1j and k). This was confirmed in PBMC when stratifying the expansions from Supplementary Fig. S1a according to HLA-A∗03:01 and HLA-DRB1∗15:01 (Supplementary Fig. S1l).

### Clinical efficacy of glatiramer acetate treatment is mediated by HLA-A∗03:01

Considering the large number of sequenced patients, the strength of the observed signal, and the performance of the ROC curves in the validation cohorts, it was surprising to see the restriction of common clone expansions to only two alleles, HLA-A∗03:01 and HLA-DRB1∗15:01. This was in clear contrast to private expansions, which can generally be observed in GA-treated patients (Supplementary Fig. S1a and l). This selectivity prompted the question whether presence of any of these two specific alleles might be associated with clinical benefits of GA. Therefore, we retrospectively analysed five cohorts of patients with MS with available clinical and HLA information, who were treated either with GA or IFN. All analysed clinical, MRI, and laboratory outcome parameters including patient numbers, effect sizes/hazard ratios, standard errors/95% confidence intervals, and p-values are summarised in Table 2.

**Table 2:** Assessment of GA efficacy with respect to HLA-A∗03:01.

Cohort	Patients	IFN A∗03−	IFN A∗03+	GA A∗03−	GA A∗03+	Outcome	Effect size (β) [±standard error]	Hazard ratio [95% CI]	p-value	Significance
ACP	54	17	11	14	12	Δ EDSS per year	−0.34 [±0.13]		0.0085^c^	∗∗
	148	61	33	39	15	Δ T2/Gd-enh./FLAIR lesions	−0.62 [±0.21]		0.0031	∗∗
BIONAT^a^	600	301	65	178	56	EDSS	−0.62 [±0.31]		0.0455	∗
						MSSS	−1.34 [±0.50]		0.0072	∗∗
CombiRx^b^	693	112	56	112 {258}	60 {95}	Relapse risk		0.66 [0.45–0.98] {0.67 [0.47–0.96]}	0.0377 {0.0269}	∗ {∗}
	563	86	44	97 {206}	50 {80}	Risk of first relapse		0.90 [0.49–1.64] {0.89 [0.52–1.50]}	0.7295 {0.6575}	n.s. {n.s.}
	604	95	49	100 {225}	49 {86}	Δ MSFC [increase indicates amelioration]	0.11 [±0.05] {0.04 [±0.04]}		0.0260 {0.3368}	∗ {n.s.}
	563	86	44	97 {206}	50 {80}	Δ EDSS per year	0.03 [±0.11] {0.04 [±0.10]}		0.7940 {0.6524}	n.s. {n.s.}
	558	84	43	98 {207}	50 {76}	Δ T1+T2 lesion volume	−2.52 [±5.79] {−4.06 [±5.28]}		0.6631 {0.4418}	n.s. {n.s.}
						CUA	−0.58 [±0.82] {−0.03 [±0.75]}		0.4803 {0.9650}	n.s. {n.s.}
EPIC	117	24	17	55	21	Δ EDSS per year	−0.34 [±0.13] {−0.42 [±0.17]}		0.0085^c^ {0.0137}	∗∗ {∗}
	125	26	19	57	23	Relapses per year	0.08 [±0.15]		0.3764	n.s.
NationMS	335	165	74	64	32	Risk of first relapse (baseline until one year follow up)		0.37 [0.16–0.88]	0.0246	∗
	40	15	14	6	5	Δ Nfl (baseline until one year follow up)	−1.35 [±0.63]		0.0410	∗
	342	173	78	65	26	Gd-enh. lesions (until one year follow up)	−2.42 [±1.62]		0.1356	n.s.
	299	140	70	60	29	Δ EDSS per year	−0.34 [±0.13] {−0.42 [±0.21]}		0.0085^c^ {0.0459}	∗∗ {∗}

As a shorthand A∗03 is used for HLA-A∗03:01. GA = glatiramer acetate, IFN = interferon beta, Δ = delta, EDSS = expanded disability status scale, Gd.-enh. = gadolinium-enhancing, FLAIR = fluid-attenuated inversion recovery, MSSS = multiple sclerosis severity score, MSFC = total multiple sclerosis functional composite, CUA = combined unique activity, β = beta coefficient from a linear (mixed) model, HR = hazard ratio from a Cox regression model [<1 indicates reduced risk, >1 indicates higher risk], n.s. = not significant, ∗ and ∗∗ = indicate p-values of below 0.05 and 0.01. Depicted p-values are not adjusted for multiple testing.

a BIONAT did not compare IFN with GA, but prior treatment with GA against no prior treatment with GA, matched for age, sex, HLA-A∗03:01 and HLA-DRB1∗15:01.

b CombiRx’ combination treatment arm (IFN + GA) provided a second set of patients and a second p-value in comparison to IFN within the cohort in addition to the GA arm. These numbers are given in brackets {}. Additionally, CombiRx only provided two-digit HLA class I so the analysis was conducted with HLA-A∗03 instead of HLA-A∗03:01.

c ACP, EPIC, and NationMS were analysed together in one model for delta EDSS per year, which resulted in one common p-value. As the number of patients was sufficiently high in EPIC and NationMS, these cohorts were also analysed on their own resulting in the p-value in brackets {}.

**Fig. 2:**
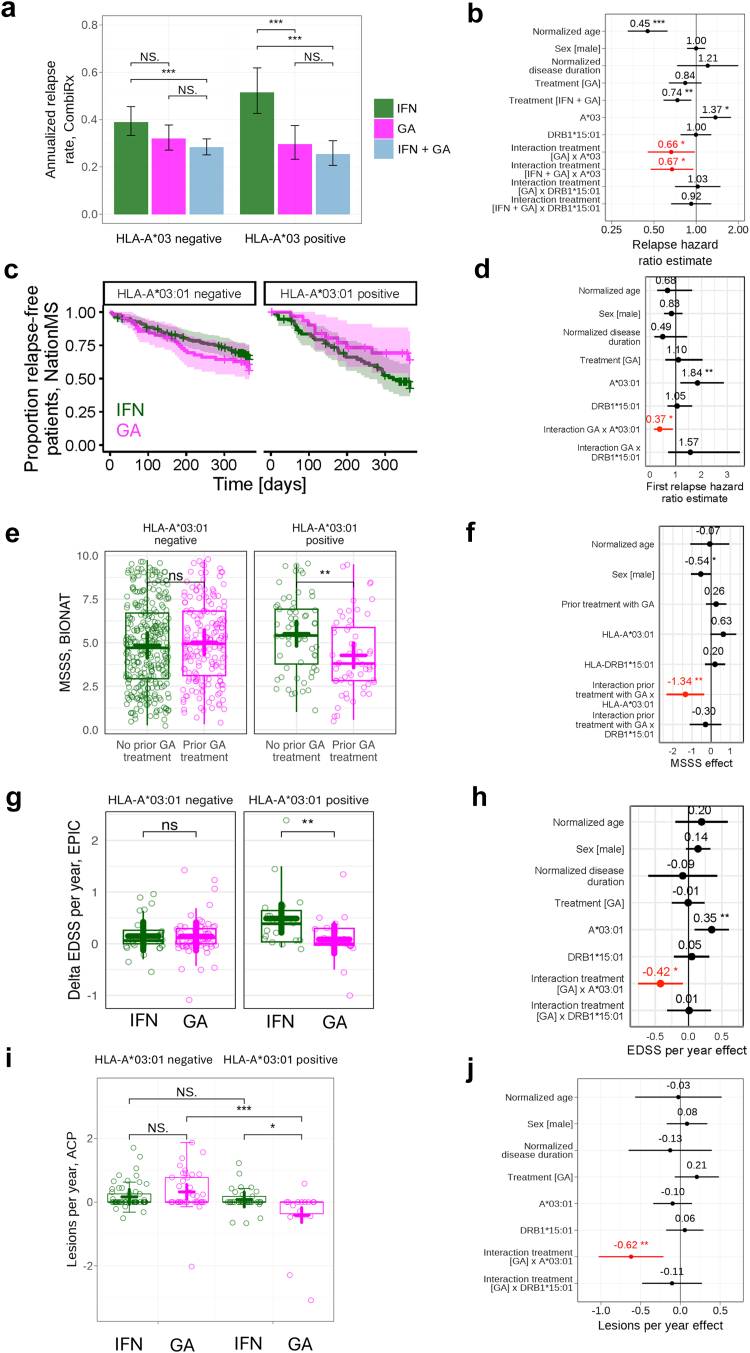
**Clinical efficacy of GA treatment is mediated by HLA-A∗03:01.** a: Annualised relapse rate in HLA-A∗03 negative (left, n(IFN) = 112, n(GA) = 112, n(IFN + GA) = 258) and HLA-A∗03 positive patients (right, n(IFN) = 56, n(GA) = 60, n(IFN + GA) = 95) of the CombiRx cohort; Error bars indicate 95% CI from a Poisson model. Statistical assessment was performed using a Cox regression model of relapse data with Andersen-Gill modification, subset to the two compared groups. HLA-A∗03 negative: p(IFN *vs.* GA) = 0.15, p(IFN *vs.* IFN + GA) = 0.00065, p(GA *vs.* IFN + GA) = 0.12. HLA-A∗03 positive: p(IFN *vs.* GA) = 0.00018, p(IFN *vs.* IFN + GA) < 0.0001, p(GA *vs.* IFN + GA) = 0.38. b: Modelling relapse data from panel a by a Cox regression with Andersen-Gill modification with the covariates normalised age, sex, disease duration, GA treatment, GA + IFN treatment, HLA-A∗03, HLA-DRB1∗15:01, and the interactions of treatment and HLA covariates yields a p-value of 0.0377 for the HLA-A∗03 GA interaction and a p-value of 0.0269 for the HLA-A∗03 IFN + GA interaction. c: Kaplan–Meier curves with proportions of relapse-free patients *vs.* observation period in days of the NationMS cohort; Left panel indicates HLA-A∗03:01 negative patients (n(IFN) = 165, n(GA) = 64), right panel HLA-A∗03:01 positive patients (n(IFN) = 74, n(GA) = 32). d: Modelling data from panel c with a Cox regression and the covariates normalised age, sex, normalised disease duration, GA treatment, HLA-A∗03:01, HLA-DRB1∗15:01, and the interactions of treatment and HLA covariates yields a p-value of 0.0246 for the HLA-A∗03:01 GA interaction. e: MSSS scores at study start of matched patients from the BIONAT cohort; Left panel indicates HLA-A∗03:01 negative patients (n(no prior GA treatment) = 301, n(prior GA treatment) = 178, p = 0.42), right panel indicates HLA-A∗03:01 positive patients (n(no prior GA treatment) = 65, n(prior GA treatment) = 56, p = 0.0026). Significance was assessed using a Wilcoxon rank-sum test. f: Modelling the data from panel e with a linear regression and the covariates normalised age, sex, prior treatment, HLA-A∗03:01, HLA-DRB1∗15:01, and the interactions of treatment and HLA covariates yields a p-value of 0.0072 for the HLA-A∗03:01 GA interaction. g: Annual change in EDSS scores of the EPIC cohort; Left panel indicates HLA-A∗03:01 negative patients (n(IFN) = 24, n(GA) = 55, p = 0.62), right panel indicates HLA-A∗03:01 positive patients (n(IFN) = 17, n(GA) = 21, p = 0.0096). Significance was assessed using a Wilcoxon rank-sum test. h: Modelling the data from panel g with a linear regression and the covariates normalised age, sex, normalised disease duration, treatment, HLA-A∗03:01, HLA-DRB1∗15:01, and the interactions of treatment and HLA covariates yields a p-value of 0.0137 for the HLA-A∗03:01 GA interaction. i: Difference in brain lesions of the ACP cohort; Left boxes indicate HLA-A∗03:01 negative patients (n(IFN) = 61, n(GA) = 39), right boxes indicate HLA-A∗03:01 positive patients (n(IFN) = 33, n(GA) = 15). Significance was assessed using a Wilcoxon rank-sum test. p(HLA-A∗03:01 negative; IFN *vs.* GA) = 0.069, p(HLA-A∗03:01 positive; IFN *vs.* GA) = 0.022, p(IFN; HLA-A∗03:01 negative *vs.* -positive) = 0.45, p(GA; HLA-A∗03:01 negative *vs.* -positive) = 0.00052. j: Modelling the data from panel i with a linear regression and the covariates normalised age, sex, normalised disease duration, treatment, HLA-A∗03:01, HLA-DRB1∗15:01, and the interactions of treatment and HLA covariates yields a p-value of 0.0031 for the HLA-A∗03:01 GA interaction. As shorthand, A∗03, A∗03:01 and DRB1∗15:01 are used to refer to HLA-A∗03, HLA-A∗03:01 and HLA-DRB1∗15:01. Green bars, boxes and lines indicate IFN treatment, magenta bars, boxes and lines GA treatment, and light blue bars indicate IFN + GA treatment; Boxes indicate the 25% and 75% percentile and median, whiskers indicate 1.5× inter-quartile range, + indicates the mean. The asterisks indicate a p-value of <0.05 (∗), <0.01 (∗∗), or <0.001(∗∗∗).

We first performed a *post-hoc* analysis of the CombiRx trial,^37^^,^^39^ where treatment-naïve patients were either treated with IFN, GA, or both for seven years (including the extension phase). For the analysis, relapses in the IFN arm were compared to the GA arm or to the IFN + GA arm. The relapse risk was significantly lower in both the GA and the IFN + GA arm compared to the IFN arm in HLA-A∗03 carriers (p_GA_ = 0.0357; p_IFN + GA_ = 0.0248; Fig. 2a and b and Supplementary Fig. S2a and b). We then assessed time to first relapse in the NationMS cohort, from treatment-naïve baseline at diagnosis to one year follow up. Compared to IFN treatment, the relapse risk was lower in GA-treated patients with MS carrying HLA-A∗03:01 (p = 0.0083; Fig. 2c and d and Supplementary Fig. S2). To rule out that the effect is only detectable in younger patients, who statistically suffer from higher relapse rates, the dataset was analysed separately in younger and older patients with comparable hazard ratios in both groups (Supplementary Table S3).

Concerning disease severity, the MS Severity Score (MSSS) and the expanded disability status scale (EDSS) were analysed cross-sectionally at baseline of the large BIONAT cohort. For this, two groups of patients (either with prior GA therapy or without) were matched for age, sex, and presence of HLA-A∗03:01, as well as HLA-DRB1∗15:01. MSSS and EDSS were lower in HLA-A∗03:01 carriers treated with GA compared to HLA-A∗03:01 carriers not previously treated with GA (p_MSSS_ = 0.0072, Fig. 2e and f; p_EDSS_ = 0.0455; Supplementary Fig. S3a and b). EDSS was then analysed longitudinally in the EPIC cohort, assessed at baseline in newly diagnosed patients with MS and then after a mean period of observation of 4.4 years on either IFN or GA treatment. Compared to IFN, the EDSS increase per year was lower in HLA-A∗03:01 carriers treated with GA (p = 0.0248; Fig. 2g and h). This was also observed in an individual analysis for NationMS baseline to one year follow up (p = 0.0399), as well as in a grouped analysis including longitudinal data from EPIC, NationMS and ACP, where EDSS was assessed on two separate occasions one to five years apart (p = 0.0074; Supplementary Fig. S3c–f).

As additional measures, patients of the ACP cohort presented with lower number of T2/Gadolinium-enhancing/FLAIR lesions during GA therapy, if they were HLA-A∗03:01 carriers (p = 0.0031; Fig. 2i and j and Supplementary Fig. S4). The total MS Functional Composite (MSFC) showed a higher increase over time, indicating amelioration, in HLA-A∗03 carrying patients in the GA arm of CombiRx (p_GA_ = 0.0146; Supplementary Fig. S5a and b). Lastly, delta neurofilament light (Nfl) was lower in HLA-A∗03:01 carriers of the NationMS cohort, from baseline to one year follow up (p = 0.0453; Supplementary Fig. S5c and d).

When directly comparing treatments within HLA-A∗03(:01) carriers, GA led to a relative reduction of relapse risk compared to IFN of 33% (CombiRx: GA + IFN arm: HR 0.67 [95% CI: 0.47–0.96], p = 0.0269) and 34% (CombiRx: GA arm: HR 0.66 [95% CI: 0.45–0.98], p = 0.0377) (Fig. 2b), and in risk to first relapse of 63% (NationMS: HR 0.37 [95% CI: 0.16–0.88], p = 0.0246) (Fig. 2d). Within HLA-A∗03:01 carriers of the BIONAT cohort, patients with prior GA treatment presented with a modelled 1.34 reduction of MSSS compared to patients without prior GA treatment (prior GA: 4.27 [95% CI: 3.69–4.86] *vs.* without prior GA: 5.51 [95% CI: 4.97–6.06, p = 0.0072; Fig. 2f). Within HLA-A∗03:01 carriers of the EPIC cohort, GA-treated patients showed a modelled reduction of EDSS worsening over time of 0.42 per year (0.08 [95% CI: −0.12 to 0.27] in GA-treated patients *vs.* 0.47 [95% CI: 0.15–0.79] in IFN-treated patients, p = 0.0137; Fig. 2h).

The analytical sequence of the study is outlined in Supplementary Fig. S6. Additional clinical assessments are shown in the Supplementary material (relapse measures: Supplementary Fig. S2; EDSS: Supplementary Fig. S3; MRI: Supplementary Fig. S4; Additional measures: Supplementary Fig. S5; All model outputs including all covariates: Supplementary Table S3). In addition to differences in the setup of the five analysed cohorts, not every measure was available for every cohort and within the cohorts, not available for every patient. HLA-DRB1∗15:01 and its interaction with GA was included in all models and did not confer positive GA effects in any of the analyses. The influence of HLA-A∗03(:01) reached significance in ten out of 16 clinical, MRI, and laboratory outcomes (Table 2). In six of the assessed cohorts of this study, between 29% and 49% of patients with MS were HLA-A∗03:01 carriers and HLA-A∗03:01 was in moderate linkage disequilibrium to HLA-DRB1∗15:01 (Table 3). For Cox regressions, Schoenfeld residuals are shown in Supplementary Table S3. All 16 analyses from Table 2 were repeated stratified by HLA-A∗03(:01) to assess potential effects of HLA-DRB1∗15(:01) in HLA-A∗03(:01) non-carriers and are also given in Supplementary Table S3.

**Table 3:** Patient numbers grouped according to HLA-A∗03:01 and HLA-DRB1∗15:01.

Cohort	Total	A∗03:01+	%	DRB1∗15:01+	%	A∗03:01+ DRB1∗15:01−	%	A∗03:01− DRB1∗15:01+	%	A∗03:01− DRB1∗15:01−	%	A∗03:01+ DRB1∗15:01+	%	Linkage disequilibrium [normalised D']
ACP	309	101	0.33	136	0.44	41	0.13	76	0.25	132	0.43	60	0.19	0.27
BIONAT	1255	359	0.29	564	0.45	147	0.12	352	0.28	544	0.43	212	0.17	0.26
EPIC	178	57	0.32	98	0.55	22	0.12	53	0.3	68	0.38	35	0.2	0.14
FinBB	1049	515	0.49	489	0.47	250	0.24	224	0.21	310	0.3	265	0.25	0.10
NationMS	405	125	0.31	193	0.48	45	0.11	113	0.28	167	0.41	80	0.2	0.31
		A∗03+		DRB1∗15:01+		A∗03+ DRB1∗15:01−		A∗03− DRB1∗15:01+		A∗03− DRB1∗15:01−		A∗03+ DRB1∗15:01+		
CombiRx	699	216	0.31	341	0.49	70	0.1	195	0.28	288	0.41	146	0.21	0.37

## Discussion

The treatment landscape of MS has opened up significantly since the introduction of GA 30 years ago. However, approximately 10% of patients with MS are still currently on therapy with GA in Germany,^40^ because this drug has one of the most favourable safety profile among approved MS drugs. Of note, GA is the only disease-modifying drug without any safety signal during pregnancy.^41^ Deciding between the different therapeutic options can be difficult for the treating physician, because personalised therapy of patients with MS is currently hampered by the lack of validated biomarkers.^16^^,^^42^ With this study we propose HLA-A∗03:01 as a directly applicable, predictive treatment response biomarker. Our data support the claim that patients with MS, who in consultation with their physician have chosen to begin a platform therapy, should be tested for HLA-A∗03:01. In patients with MS of European ancestry, between 29% (BIONAT) and 49% (FinBB) were HLA-A∗03:01 positive. These positive patients should be treated with GA over IFN while considering other factors such as allergies or personal preference.

While previous studies could not identify any association between HLA alleles and clinical response to IFN,^22^^,^^43^, ^44^, ^45^ there have been prior studies suggesting a moderate effect of the HLA-DRB1∗15 - HLA-DQB1∗06 haplotype in association with a more favourable GA response.^22^, ^23^, ^24^ However, the studies regarding GA focused on HLA class II and their results may have been influenced by the moderate linkage disequilibrium of HLA-DRB1∗15:01 and HLA-A∗03:01.^18^ We could show that in our stratification analyses there was no influence of HLA-DRB1∗15:01 in patients without HLA-A∗03:01, which supports the notion that the previously published HLA class II findings might have been influenced by this statistical over-representation. This is especially interesting, because these two linked backgrounds were the only HLA alleles with a pattern of multiple GA-associated TRBs. In line with our findings, indications of a putatively common, HLA-I-restricted response to GA has been observed before with one published TRB sequence (CASSLGQGAGGYT) being one Hamming distance away from one newly detected, HLA-A∗03:01-associated sequence CASSSGQGAGGYTF including the same GA-associated motif GQGA.^46^

Based on this finding, there is a need for future studies. As HLA-A∗03:01 carriers respond more favourably to GA, it could be argued that GA might also outperform treatments other than IFN in these patients. The relative reduction in relapse risk of 33%–66% depending on the cohort compared to IFN would put GA at least in the range of dimethyl fumarate and fingolimod in genetic GA responders, potentially even in the range of high-efficacy therapies according to meta-analyses.^6^ It would be important to conduct systematic post-hoc analyses of the large clinical trials to directly compare these therapies to GA in HLA-A∗03:01 carriers.

The MOA of the HLA-A∗03:01-associated GA-expanded CD8+ T cells remains an open question. While it seems obvious, if unproven *in vitro*, that these cells react to the drug, GA specificity alone would not explain their function in the context of MS. It is conceivable that these cells recognise more than just GA and that this secondary target might be the reason why GA specifically works in HLA-A∗03:01 positive patients. If future approaches can use the paired single-cell T-cell receptor sequences from the current study to elucidate what the secondary target(s) might be, this would be informative about MS pathology and could potentially be the basis for an improved formulation of peptides that might also be beneficially presented in other HLA backgrounds. Additionally, if the beneficial peptide(s) that confer the clinical effects in HLA-A∗03:01 carriers could be determined from the mixture of GA, which includes immense numbers of random epitopes, isolated doses of these peptides might also be given to focus the immune response and potentially enhance GA’s efficacy in responders. One interesting aspect of the TRB patterns is the strong presence of the variable beta chain TRBV5-6, even though the HLA allele usually strongly determines selection of variable beta chains^47^ and it is rather unlikely that two (or more) alleles (MHC class I and –II) would lead to selection of a common, usually low-expressed variable chain by chance. This could mean that there is similarity and possibly some cross-presentation of the same epitope(s), which would also be supported by the finding that the expansion of HLA-A∗03:01-associated GA clones was boosted by the additional presence of HLA-DRB1∗15:01 in patients. The fact that both analysed alleles expand public T-cell clones, but only the more focused HLA-A∗03:01 expanded CD8+ T cells lead to a detectable clinical benefit should also be a topic of future studies concerning the role of different T-cell lineages in MS pathology.

Not all clinical and MRI outcomes reached significance in all cohorts. While some of this can be attributed to different statistical power due to different numbers of patients for each measure, it could also suggest that GA has a more consistent and stronger effect on some clinical aspects than others. This would be in line with results of the REGARD trial, where there was no difference between IFN and GA in overall efficacy, but IFN showed a stronger effect on reduction of gadolinium-enhancing lesions, whereas GA had a stronger influence on reduction of brain atrophy.^5^^,^^8^

There are some caveats to this study. It was observed that without GA, overall disease activity was higher in HLA-A∗03:01 carriers of most cohorts and future studies are needed to assess whether HLA-A∗03:01 might contribute to worse disease prognosis in patients with MS under treatments other than IFN. It has been shown that HLA-A∗03:01-restricted T-cell clones can cause MS-like disease in a humanised animal model, which was mitigated by the protective HLA-A∗02:01 allele.^17^ Influence of HLA-A∗03:01 on MS severity has not been tested in the largest study to date,^19^ most likely due to the fact that the allele is only associated with higher MS incidence due to its LD with HLA-DRB1∗15:01. For the CombiRx trial, apart from relapse data to be consistent with the original publication,^37^ we analysed longitudinal measures at the three-year mark before the start of the extension phase to exclude a possible selection bias from patient dropout. It is possible that the interaction effect is strengthened at longer times, and indeed trends in EDSS and MSFC were supportive in this direction, but this would require a longer study period to formally ascertain. It might also be possible that IFN reduces the GA effect in genetic responders of the combination treatment arm, because IFN can induce mild lymphopenia,^48^ which might interfere with successful establishment of beneficial T-cell expansions. Additionally, the interaction effect between GA and HLA-A∗03 in CombiRx was likely underestimated due to the fact that only two-digit HLA information was available. The HLA-A∗03:01 pattern is not visible in the <1% of HLA-A∗03:02 positive patients. There is also the possibility that other HLA alleles might confer some GA effects, but that these effects were either too weak to detect or present in too few patients to detect reliably. This is an important point, because we cannot claim without placebo data that GA does not work at all in HLA-A∗03:01 negative patients, only that it works significantly better in positive patients compared to negative patients and thereby outperforms IFN in positive patients. Whether there is no effect at all or some residual effect in negative patients is unclear, but probably immaterial, as these patients would benefit more from IFN and choose it over GA. Of note, there were also some positive homozygosity effects in the cohorts, where that information was available (NationMS and CombiRx), but with the missing information in other cohorts, we decided to not analyse this further. That being said, there is a high chance that HLA-A∗03:01 homozygous patients benefit particularly well from GA, which would, of course, be important information for the future comparison with other drugs. As our study cohorts consisted mostly of patients with a European genetic background, it is possible that GA might confer its effects differently in patients of other ethnicities.^49^ A last but important point is the retrospective approach of our study. A prospective trial within HLA-A∗03:01 carriers with different treatment options would be an ideal approach to address many of these questions.

Taken together, these data, derived from one mono- and four multi-centric cohorts of 1007 GA-treated patients with MS, strongly suggests clinical utility in testing for the presence of the HLA-A∗03:01 allele prior to starting a platform therapy. Our data suggest that GA therapy would prove preferable over IFN in HLA-A∗03:01 carriers. Future studies will have to show whether GA might also outperform other therapies than IFN in these carriers and what this finding might mean for MS pathology and the potential design of improved therapies.

## Contributors

Conceptualisation: BCZ, TSH, RE, DMX, BC, JO, MRW, SLH, SZ, LK, RL, JJS, HW, NS.

Data curation: BCZ, TSH, RE, BP, SF, CW, MD, DM, DMX, ED, LAG, FB, LR, GA, SJ, CS, MS, SB, FL, JH, MKN, MF, SR, AS, FH, RH, SC, ASANT, MSEIP, CH, SN, AB, HAT, FTB, GMZH, TK, CCG, BW, MK, RG, SGM, FZ, BC, JO, MRW, SLH, SZ, LK, RL, JJS, HW, NS.

Formal analysis: BCZ, TSH, RE, DM, DMX, NS.

Funding acquisition: JO, MRW, SLH, JJS, NS.

Investigation: BCZ, TSH, RE, DM, DMX, JO, SZ, LK, RL, JJS, NS.

Methodology: BCZ, RE, NS.

Supervision: LAG, SB, AS, CCG, BW, MK, RG, SGM, FZ, BC, JO, MRW, SLH, SZ, LK, RL, HR, JJS, HW, NS.

Validation: BCZ, TSH, JO, SZ, LK, JJS, HW, NS.

Visualisation: BCZ, TSH, RE, NS.

Writing – original draft: BCZ, TSH, RE, DM, JJS, NS.

Writing– review & editing: BCZ, TSH, RE, BP, SF, CW, MD, EY, DM, DMX, ED, LAG, FB, LR, GA, SJ, CS, MS, SB, FL, JH, MKN, MF, SR, AS, FH, RH, SC, ASANT, MSEIP, CH, SN, AB, HAT, FTB, GMZH, TK, CCG, BW, MK, RG, SGM, FZ, BC, JO, MRW, SLH, SZ, LK, RL, HR, JJS, HW, NS.

Both study groups contributed to patient recruitment and data curation.

Brian Zhang, Tilman Schneider-Hohendorf, Rebecca Elyanow and Nicholas Schwab have accessed the complete underlying data and have verified the analyses. All authors read and approved the final version of the manuscript.

## Data sharing statement

The GA-associated TRB sequences and the single-cell RNA sequencing TCR alpha and beta chain sequences are provided in Supplementary Tables S1 and S2, the single-cell RNA sequencing transcriptomics data with TCR sequences are shared openly on Zenodo 10.5281/zenodo.15772651. The clinical trial data must be requested at the respective offices.

## Declaration of interests

BCZ: is an employee of Adaptive Biotechnologies and receives salary, stock, and options as part of their employment compensation outside of the submitted work.

TSH: reports travel support from Roche and Biogen, research support from Novartis Pharma and is co-inventor on patents outside of this study.

RE: is an employee of Adaptive Biotechnologies and receives salary, stock, and options as part of their employment compensation outside of the submitted work.

BP: nothing to disclose.

SF: nothing to disclose.

CW: nothing to disclose.

MD: nothing to disclose.

EY: is an employee of Adaptive Biotechnologies and receives salary, stock, and options as part of their employment compensation outside of the submitted work.

DM: is an employee of Adaptive Biotechnologies and receives salary, stock, and options as part of their employment compensation outside of the submitted work.

DMX: is an employee of Adaptive Biotechnologies and receives salary, stock, options and patent participation as part of their employment compensation, outside of the submitted work.

ED: nothing to disclose.

LAG: received research funding from Hertie Foundation (funding of MS TWIN STUDY + personal funding), from DMSG national and Bavarian section (funding of MS TWIN STUDY), and from the DFG for the SyNergy Cluster (funding of MS TWIN STUDY + personal funding).

FB: nothing to disclose.

LR: nothing to disclose.

GA: nothing to disclose.

SJ: nothing to disclose.

CS: nothing to disclose.

MS: reported consulting fees from Alexion, Amgen/Horizon, Bayer, Biogen, Bristol-Myers-Squibb/Celgene, Merck, Roche, and Sanofi Genzyme, honoraria from Alexion, Janssen, Amgen/Horizon, Biotest, Roche, Sanofi Genzyme, and UCB, travel support from Alexion, Celgene, Janssen, Amgen/Horizon, Roche, and Sanofi Genzyme, and participation on advisory boards for Alexion, Amgen/Horizon, Bayer, Biogen, Bristol-Myers-Squibb, Merck, Roche, and Sanofi Genzyme.

SB: received honoraria from Biogen, Bristol Myers Squibb, Hexal, Merck Healthcare, Novartis, Roche, Sanofi and Teva. He is supported by the Deutsche Forschungsgemeinschaft (DFG, SFB CRC TRR 355–480846870), Novartis and the Hermann- and Lilly-Schilling Foundation.

FL: received consultancy fees from Roche and support with travel cost from Teva Pharma.

JH: reported grants for OCT research from the Sumaira Foundation, Horizon/Amgen, Roche and Merck, personal consulting fees and honoraria from Alexion, Amgen, Merck, Novartis, Neuraxpharm, Johnson&Johnson, Rewind Therapeutics and Roche and nonfinancial support of the Sumaira-Foundation and Guthy-Jackson Charitable Foundation, all outside the submitted work.

MKN: nothing to disclose.

MF: nothing to disclose.

SR: nothing to disclose.

AS: received speaker honoraria from Bristol Myers Squibb, Merck, Neuraxpharm, Novartis, consulting fees from Neuraxpharm, and research support by the Medical Faculty of the University of Bern, the Swiss MS Society and the regional association of North Rhine-Westphalia of the German MS Society (DMSG Landesverband NRW) and Novartis, all not related to this work.

FH: received grants or contracts from Japan Society for the Promotion of Science (JSPS, Overseas Research Fellowship, 2023-present), the Uehara Memorial Foundation (Overseas Research Fellowship, 2022–2023), and the Japanese Society of Neurology (Overseas Training Program, 2022–2023), outside this study.

RH: his institution received grants from Roche/Genentech and he received consulting fees from Roche, Sanofi and Novartis, lecture honoraria from Sanofi, and compensation for participation on advisory boards from Roche, Novartis and Sanofi.

SC: nothing to disclose.

ASANT: nothing to disclose.

MSEIP: reported speaker honoraria from Merck, Novartis and Roche as well as travel support from AB Science, Alkermes, Baxalta, Bayer, Biogen, CSL Behring, Genzyme, Griffols, Merck, Novartis, Octapharma, Receptoc, Roche, Sanofi Aventis, Teva, UCB Biopharma.

CH: nothing to disclose.

SN: received lecture honoraria from Roche, Novartis, and Sanofi, travel support from Sanofi, and received compensation for serving on a scientific advisory board of Roche, Neuraxpharm, Bristol –Myers Squibb and Merck.

AB: received consulting fees from Roche, Merck, Novartis, Sandoz/HEXAL, lecture honoraria from Merck, Biogen, Novartis, TEVA, Roche, Sanofi/Genzyme, Bristol Myers Squibb, Jannsen, and Sandoz/HEXAL, and received travel support from Biogen, TEVA, Novartis, Sanofi/Genzyme, Merck, Celgene and Jannsen.

HAT: reported unpaid roles in DGLN, DGN, DMSG, AMSEL, KKNMS, DGG, Pflegebrücke, Albert-Einstein-Discovery centre e.V., personal consulting fees or lecture honoraria from Alexion, Bayer, Biogen, Bristol-Myers Squibb, Fresenius, Fujirebio, Hexal, Horizon, Janssen, Johnson&Johnson, Merck, Novartis, Roche, Sanofi, Teva, Viatris, and grants from DMSG, Ministerium für Wissenschaft und Forschung BW, Novartis, Sanofi, Faber-Stiftung, Chemische Fabrik Karl Bucher GmbH to his institution outside the submitted work.

FTB: received grants or contracts from German Science Fund (DFG), German Federal Ministry of Education and Science (BMBF), Diamed and Fresenius, consulting fees from Amgen-Horizon, Biogen, Merck, Novartis, Roche, Sanofi-Genzyme and lecture honoraria from Alexion, Amgen-Horizon, Fresenius, Merck, Novartis, Roche, Takeda, travel support from Fresenius, Merck, Neuraxpharm, Roche, Sanofi-Genzyme and Teva.

GMZH: was supported by grants from the Deutsche Forschungsgemeinschaft (DFG) (ME4050/12–1, ME4050/13–1), a grant from the Bundesministerium für Bildung und Forschung (BMBF) ‘Lipid Immune Neuropathy Consortium’ grants from Roche and from Novartis. GMzH has received speaker honoraria/travel support from Alexion, LFB Pharma, Argenx, Janssen. He has received reimbursement for serving on AdBoards/Trial Steering Comittees from LFB Pharma, Roche, Immunovant, Argenx. He has received project-related research funding from Merck KGaA, Biogen, Roche and material support from Novartis.

TK: received speaker honoraria and/or personal fees for advisory boards from Novartis Pharma, Roche Pharma, Alexion/Astra Zeneca, Horizon Therapeutics/Amgen, Merck, Chugai Pharma and Biogen. The Institution she works for has received compensation for serving as a member of a steering committee from Roche. TK is a site principal investigator in several randomised clinical trials (Novartis Pharma, Roche Pharma, BMS and Sanofi Genzyme) and in a randomized clinical trials supported by the BMBf (funding code: 01GM1908E) and her institution has received compensation for clinical trials all outside the present work. TK is Part of the managing board of the German TK Part of the managing board of the German “krankheitsbezogenes Kompetenznetz Multiple Sklerose” (KKNMS) and of the German Neuromyelitis Optica Study Group (NEMOS) – both unpaid.

CCG: received grants from European Joint Programme on Rare Diseases (EJP RD), Horizon 2020 ReSToRE, Biogen, Roche, Sanofi, licence fees for European patent No. 4314829, lecture honoraria from the DIU Dresden International University GmbH. She issued a patent with the number EP22199707.5 and is unpaid extended board member and member of the Education Commission of the DGLN e.V., member of the Advisory Board of the DAkkS and board member of EUSAC e.V.

BW: reported grants from the Deutsche Forschungsgemeinschaft, German Ministry of Education and research, Baden-Württemberg Ministry for Science, Research and Art, Dietmar Hopp Foundation, Klaus Tschira Foundation, grants and personal fees from Merck, ArgenX, Novartis, and personal fees from Alexion, INSTAND, Roche.

MK: received speaker honoraria and/or personal fees for advisory boards from Biogen, Janssen-Cilag, Merck, Novartis, Sanofi, Roche and Teva as well as grant support from Sanofi and Roche.

RG: received research support from Teva Pharmaceutical Industries, Biogen Idec, Bayer Schering Pharma, Genzyme, Merck Serono, and Novartis, speaker honoraria from Biogen Idec, Teva Pharmaceutical Industries, Bayer Schering Pharma, and Novartis and participated on scientific advisory boards for Teva Pharmaceutical Industries, Biogen Idec, Bayer Schering Pharma, Kyverna, and Novartis. RG is an editorial board member of Therapeutic Advances in Neurological Diseases and Experimental Neurology and The Journal of Neuroimmunology.

SGM: received honoraria for lecturing, travel expenses and for attending meetings from Academy 2, Argenx, Alexion, Almirall, Amicus Therapeutics Germany, AstraZeneca, Bayer Health Care, Biogen, BioNtech, BMS, Celgene, Datamed, Demecan, Desitin, Diamed, Diaplan, DIU Dresden, DPmed, Gen Medicine and Healthcare products, Genzyme, Hexal AG, IGES, Impulze GmbH, Janssen Cilag, KW Medipoint, MedDay Pharmaceuticals, Medmile, Merck Serono, MICE, Mylan, Neuraxpharm, Neuropoint, Novartis, Novo Nordisk, ONO Pharma, Oxford PharmaGenesis, QuintilesIMS, Roche, Sanofi, Springer Medizin Verlag, STADA, Chugai Pharma, Teva, UCB, Viatris, Wings for Life international and Xcenda. His research is funded by the German Ministry for Education and Research (BMBF), German Federal Institute for Risk Assessment (BfR), German Research Foundation (DFG), Else Kröner Fresenius Foundation, Gemeinsamer Bundesausschuss (G-BA), German Academic Exchange Service, Hertie Foundation, Interdisciplinary Centre for Clinical Studies (IZKF) Muenster, German Foundation Neurology, Ministry of Culture and Science of the State of North Rhine-Westphalia, The Daimler and Benz Foundation, Multiple Sclerosis Society North Rhine-Westphalia Regional Association (dmsg), Peek & Cloppenburg Düsseldorf Foundation, Hempel Foundation for Science, Art and Welfare, German Alzheimer Society e.V. Dementia self-help and Alexion, Almirall, Amicus Therapeutics Germany, Argenx, Bayer Vital GmbH, BGP Products Operations (Viatris Company), Biogen, BMS, Demecan, Diamed, DGM e. v., Fresenius Medical Care, Genzyme, Gesellschaft von Freunden und Förderern der Heinrich-Heine-Universität Düsseldorf e.V., HERZ Burgdorf, Hexal, Janssen, Merck Serono, Novartis, Novo Nordisk Pharma, ONO Pharma, Roche and Teva.

FZ: received consultation funds from: Amgen, Biogen, Max Planck Society (MPG), Bristol-Meyers-Squibb, Celgene, Hexal, Horizon, Janssen, Merck Serono, Novartis, Roche, Sanofi Genzyme, Sandoz, TEVA. Her research is funded by the DFG (CRC 1292 – 318346496) and BMBF VIP + CheckAut.

BC: reported grants or contracts from Genentech and Kyverna to UCSF, consulting fees from Alexion, Alumis, Avotres, Biogen, Boston Pharma, EMD Serono, Hexal/Sandoz, Horizon, Immunic AG, Kyverna, Neuron23, Novartis, Sanofi, Siemens and TG Therapeutics, participation on a data safety monitoring board or advisory board from IDMC – vidofludimus calcium in MS (Immunic AG).

JO: nothing to disclose.

MRW: received unrelated research grant funding from Roche/Genentech and Kyverna Therapeutics, previously received unrelated research grant funding from Novartis, has received licencing fees from CDI Labs and consulting fees and stock from Delve Bio, where he is also a board member.

SLH: currently serves on the scientific advisory boards of Accure, Alector, Hinge Bio; previously consulted for BD, Gilead, Moderna, NGM Bio, Nurix Therapeutics, Pheno Therapeutics; previously served on the Board of Directors of Neurona and currently serves as an advisor. Dr. Hauser also has received nonfinancial support (travel reimbursement and writing support for anti-CD20-therapy-related meetings and presentations) from F. Hoffmann-La Roche and Novartis AG;

SZ: nothing to disclose.

LK: received compensation for participation on data safety monitoring or advisory boards from Alexion, Biogen, Bristol-Myers Squibb, Hexal, Horizon, Janssen, Merck Serono, Novartis, Roche, Sandoz, Sanofi, Teva and Viatris, and research grants from Amgen, Argenx, Bayer, Biogen, Bristol-Myers Squibb, Grifols, Horizon, Merck Serono, Novartis, Roche, Sanofi, Santhera and Teva, as well as from the German Research Foundation, IZKF Münster, IMF Münster, all outside the submitted work.

RL: reported grants from Roche, PopulationBio and COUR-Pharma, consulting fees and materials from Novartis, and speaker honoraria from Biogen, Sanofi-Genzyme, Merck-Serono and Hikma, all outside the submitted work.

HR: is an employee of Adaptive Biotechnologies and receives salary, stock, and options as part of their employment compensation outside of the submitted work.

JJS: has previously received research grant funding from Roche/Genentech and Novartis and advisory board honoraria from IgM Biosciences and TG Therapeutics.

HW: reported consulting fees from Biogen, Merck, Novartis, Sanofi, Genzyme, UCB, Roche, Samsung, Bristol-Myers Squibb, Alexien, Argenx, Immunic, Janssen, LTS, Lundbeck, Medix Europa, Muna Therapeutics, Myrobalan Therapeutics, Peervoice, PSL Group Services, Red Nucleus, Sangamo, Syneos, Toleranzia, Viatris, Teladoc, Swiss Multiple Sclerosis Society, travel support from Biogen, Merck, Sanofi, Novartis, Teva, Springer, Streamed Up, EPG Health, WebMD Health, Ology Medical Education, participation on advisory boards for Biogen, Roche, Novartis, Merck, Genzyme, Argenx, Galapagos, UniQURE, Sandoz, Janssen Pharmaceuticals and serves as expert advisor for Biogen, Roche, Novartis, Merck, Genzyme, Argenx, Galapagos, UniQURE, Sandoz, Janssen Pharmaceuticals, all outside of the submitted work.

NS: reported grants from DFG and Roche during the conduct of the study.

## Appendix A Supplementary data

EBIOM-D-25-02650R1 Study groups

## References

[1] Woo M.S., Engler J.B., Friese M.A. (2024). The neuropathobiology of multiple sclerosis. Nat Rev Neurosci.

[2] Johnson K.P., Brooks B.R., Cohen J.A. (1995). Copolymer 1 reduces relapse rate and improves disability in relapsing-remitting multiple sclerosis: results of a phase III multicenter, double-blind, placebo-controlled trial. Neurology.

[3] Comi G., Filippi M., Wolinsky J.S., Group EGAS (2001). European/Canadian multicenter, double-blind, randomized, placebo-controlled study of the effects of glatiramer acetate on magnetic resonance imaging–measured disease activity and burden in patients with relapsing multiple sclerosis. Ann Neurol.

[4] Comi G., Martinelli V., Rodegher M. (2009). Effect of glatiramer acetate on conversion to clinically definite multiple sclerosis in patients with clinically isolated syndrome (PreCISe study): a randomised, double-blind, placebo-controlled trial. Lancet.

[5] Mikol D.D., Barkhof F., Chang P. (2008). Comparison of subcutaneous interferon beta-1a with glatiramer acetate in patients with relapsing multiple sclerosis (the REbif vs Glatiramer Acetate in relapsing MS disease [REGARD] study): a multicentre, randomised, parallel, open-label trial. Lancet Neurol.

[6] Samjoo I.A., Worthington E., Drudge C. (2021). Efficacy classification of modern therapies in multiple sclerosis. J Comp Eff Res.

[7] Liblau R. (2009). Glatiramer acetate for the treatment of multiple sclerosis: evidence for a dual anti-inflammatory and neuroprotective role. J Neurol Sci.

[8] Prod’homme T., Zamvil S.S. (2019). The evolving mechanisms of action of glatiramer acetate. Cold Spring Harb Perspect Med.

[9] Teitelbaum D., Milo R., Arnon R., Sela M. (1992). Synthetic copolymer 1 inhibits human T-cell lines specific for myelin basic protein. Proc Natl Acad Sci USA.

[10] Miller A., Shapiro S., Gershtein R. (1998). Treatment of multiple sclerosis with Copolymer-1 (Copaxone®): implicating mechanisms of Th1 to Th2/Th3 immune-deviation. J Neuroimmunol.

[11] Hong J., Li N., Zhang X., Zheng B., Zhang J.Z. (2005). Induction of CD4+CD25+ regulatory T cells by copolymer-I through activation of transcription factor Foxp3. Proc Natl Acad Sci USA.

[12] Weber M.S., Prod’homme T., Youssef S. (2007). Type II monocytes modulate T cell–mediated central nervous system autoimmune disease. Nat Med.

[13] Tennakoon D.K., Mehta R.S., Ortega S.B., Bhoj V., Racke M.K., Karandikar N.J. (2006). Therapeutic induction of regulatory, cytotoxic CD8+ T cells in multiple Sclerosis1. J Immunol.

[14] Tumani H., Kassubek J., Hijazi M. (2011). Patterns of Th1/Th2 cytokines predict clinical response in multiple sclerosis patients treated with Glatiramer acetate. Eur Neurol.

[15] Tacke S., Braune S., Rovituso D.M. (2021). B-Cell activity predicts response to Glatiramer acetate and interferon in relapsing-remitting multiple sclerosis. Neurol Neuroimmunol Neuroinflamm.

[16] Paul A., Comabella M., Gandhi R. (2019). Biomarkers in multiple sclerosis. Cold Spring Harb Perspect Med.

[17] Friese M.A., Jakobsen K.B., Friis L. (2008). Opposing effects of HLA class I molecules in tuning autoreactive CD8+ T cells in multiple sclerosis. Nat Med.

[18] International Multiple Sclerosis Genetics Consortium, Wellcome Trust Case Control Consortium 2, Sawcer S. (2011). Genetic risk and a primary role for cell-mediated immune mechanisms in multiple sclerosis. Nature.

[19] Harroud A., Stridh P., McCauley J.L. (2023). Locus for severity implicates CNS resilience in progression of multiple sclerosis. Nature.

[20] Fogdell-Hahn A., Ligers A., Grønning M., Hillert J., Olerup O. (2000). Multiple sclerosis: a modifying influence of HLA class I genes in an HLA class II associated autoimmune disease. Tissue Antigens.

[21] Harbo H.F., Lie B.A., Sawcer S. (2004). Genes in the HLA class I region may contribute to the HLA class II-associated genetic susceptibility to multiple sclerosis. Tissue Antigens.

[22] Fusco C., Andreone V., Coppola G. (2001). HLA-DRB1∗1501 and response to copolymer-1 therapy in relapsing-remitting multiple sclerosis. Neurology.

[23] Gross R., Healy B.C., Cepok S. (2011). Population structure and HLA DRB1 1501 in the response of subjects with multiple sclerosis to first-line treatments. J Neuroimmunol.

[24] Dhib-Jalbut S., Valenzuela R.M., Ito K., Kaufman M., Ann Picone M., Buyske S. (2013). HLA DR and DQ alleles and haplotypes associated with clinical response to glatiramer acetate in multiple sclerosis. Mult Scler Relat Disord.

[25] Outteryck O., Ongagna J.C., Brochet B. (2014). A prospective observational post-marketing study of natalizumab-treated multiple sclerosis patients: clinical, radiological and biological features and adverse events. The BIONAT cohort. Eur J Neurol.

[26] University of California, San Francisco MS-EPIC Team, Cree B.A.C., Gourraud P.-A. (2016). Long-term evolution of multiple sclerosis disability in the treatment era. Ann Neurol.

[27] Gittelman R.M., Lavezzo E., Snyder T.M. (2022). Longitudinal analysis of T-cell receptor repertoires reveals shared patterns of antigen-specific response to SARS-CoV-2 infection. JCI Insight.

[28] Schneider-Hohendorf T., Gorlich D., Savola P. (2018). Sex bias in MHC I-associated shaping of the adaptive immune system. Proc Natl Acad Sci USA.

[29] Carlson C.S., Emerson R.O., Sherwood A.M. (2013). Using synthetic templates to design an unbiased multiplex PCR assay. Nat Commun.

[30] Hao Y., Hao S., Andersen-Nissen E. (2021). Integrated analysis of multimodal single-cell data. Cell.

[31] Ostkamp P., Deffner M., Schulte-Mecklenbeck A. (2022). A single-cell analysis framework allows for characterization of CSF leukocytes and their tissue of origin in multiple sclerosis. Sci Transl Med.

[32] Ho D., Imai K., King G., Stuart E.A. (2011). MatchIt: nonparametric preprocessing for parametric causal inference. J Stat Softw.

[33] Storey J.D., Tibshirani R. (2003). Statistical significance for genomewide studies. Proc Natl Acad Sci USA.

[34] Begley C.G., Ellis L.M. (2012). Raise standards for preclinical cancer research. Nature.

[35] Zahid H.J., Taniguchi R., Ebert P. (2024). Large-scale statistical mapping of T-cell receptor β sequences to human leukocyte antigens. bioRxiv.

[36] Emerson R.O., DeWitt W.S., Vignali M. (2017). Immunosequencing identifies signatures of cytomegalovirus exposure history and HLA-mediated effects on the T cell repertoire. Nat Genet.

[37] Lublin F.D., Cofield S.S., Cutter G.R. (2017). Long-term follow-up of a randomized study of combination interferon and glatiramer acetate in multiple sclerosis: efficacy and safety results up to 7 years. Mult Scler Relat Disord.

[38] Klotz L., Eschborn M., Lindner M. (2019). Teriflunomide treatment for multiple sclerosis modulates T cell mitochondrial respiration with affinity-dependent effects. Sci Transl Med.

[39] Lublin F.D., Cofield S.S., Cutter G.R. (2013). Randomized study combining interferon and glatiramer acetate in multiple sclerosis. Ann Neurol.

[40] Goereci Y., Ellenberger D., Rommer P. (2024). Persons with multiple sclerosis older than 55 years: an analysis from the German MS registry. J Neurol.

[41] Bast N., Dost-Kovalsky K., Haben S. (2025). Impact of disease-modifying therapies on pregnancy outcomes in multiple sclerosis: a prospective cohort study from the German multiple sclerosis and pregnancy registry. Lancet Reg Health Eur.

[42] Comabella M., Sastre-Garriga J., Montalban X. (2016). Precision medicine in multiple sclerosis: biomarkers for diagnosis, prognosis, and treatment response. Curr Opin Neurol.

[43] Comabella M., Fernández-Arquero M., Río J. (2009). HLA class I and II alleles and response to treatment with interferon-beta in relapsing–remitting multiple sclerosis. J Neuroimmunol.

[44] Fernández O., Fernández V., Mayorga C. (2005). HLA class II and response to interferon-beta in multiple sclerosis. Acta Neurol Scand.

[45] Villoslada P., Barcellos L.F., Rio J. (2002). The HLA locus and multiple sclerosis in Spain. Role in disease susceptibility, clinical course and response to interferon-β. J Neuroimmunol.

[46] Biegler B.W., Yan S.X., Ortega S.B., Tennakoon D.K., Racke M.K., Karandikar N.J. (2006). Glatiramer acetate (GA) therapy induces a focused, oligoclonal CD8+ T-cell repertoire in multiple sclerosis. J Neuroimmunol.

[47] Marrack P., Scott-Browne J.P., Dai S., Gapin L., Kappler J.W. (2008). Evolutionarily conserved amino acids that control TCR-MHC interaction. Annu Rev Immunol.

[48] Rieckmann P., O’Connor P., Francis G.S., Wetherill G., Alteri E. (2004). Haematological effects of interferon-beta-1a (Rebif) therapy in multiple sclerosis. Drug Saf.

[49] Hollenbach J.A., Oksenberg J.R. (2015). The immunogenetics of multiple sclerosis: a comprehensive review. J Autoimmun.

