# Supplementary material for: HLA-A∗03:01 as predictive genetic biomarker for glatiramer acetate treatment response in multiple sclerosis: a retrospective cohort analysis
Source: eBioMedicine. 2025 Jul 31;118:105873. doi: 10.1016/j.ebiom.2025.105873 (PMC12336691; doi:10.1016/j.ebiom.2025.105873)
Supplement: EBIOM-D-25-02650R1 Study groups [file mmc1.docx]

Study groups:

KKNMS (NationMS) study group:

Orhan Aktas, Antje Giede-Jeppe, Barbara Gisevius, Markus Kowarik, Friedemann Paul, Veit Rothhammer, Corinna Trebst, Uwe Zettl

University of California San Francisco MS-EPIC Team:

Jessa Alexander, Riley Bove, Sergio Baranzini, Bruce A C Cree, Eduardo Caverzasi, Richard Cuneo, Stacy J Caillier, Tiffany Cooper, Ari J Green, Chu-Yueh Guo, Jeffrey M Gelfand, Refujia Gomez, Sasha Gupta, Jill Hollenbach, Meagan Harms, Roland G Henry, Stephen L Hauser, Myra Mendoza, Jorge R Oksenberg, Nico Papinutto, Sam Pleasure, Adam Santaniello, Joseph J Sabatino Jr, William A Stern, Michael R Wilson, Scott Zamvil
