# Supplementary material for: HLA-A∗03:01 as predictive genetic biomarker for glatiramer acetate treatment response in multiple sclerosis: a retrospective cohort analysis
Source: eBioMedicine. 2025 Jul 31;118:105873. doi: 10.1016/j.ebiom.2025.105873 (PMC12336691; doi:10.1016/j.ebiom.2025.105873)
Supplement: Supplementary Figs. S1–S6 and Table S1 [file mmc2.pdf]

Supplementary material

Table of contents:

**Table of contents:**	1
Table S1 with legend	2-3
Table S2 legend	3
Table S3 legend	4
Figure S1	5
Figure S1 legend	6
Figure S2	7
Figure S2 legend	8
Figure S3	9
Figure S3 legend	10
Figure S4	11
Figure S4 legend	12
Figure S5	13
Figure S5 legend	14
Figure S6	15
Figure S6 legend	16

Table S1: GA TRB sequences and -motifs, with HLA associations

HLA (p< 0.0001)	HLA (p < 0.001)	CDR3beta	Vbeta	Jbeta	Motif
A*03:01	A*03:01	CASSEDRRDYNEQFF	V25-01	J02-01	CASSEDRRxYNEQFF+V25-01
A*03:01	A*03:01	CASSEDRRNYNEQFF	V25-01	J02-01	CASSEDRRxYNEQFF+V25-01
A*03:01	A*03:01	CASSEDRRSYNEQFF	V25-01	J02-01	CASSEDRRxYNEQFF+V25-01
A*03:01	A*03:01	CASSEDRRTYNEQFF	V25-01	J02-01	CASSEDRRxYNEQFF+V25-01
A*03:01	A*03:01	CASSLSNAYEQYF	V11-02	J02-07	CASSLSNxYEQYF+V11-02
A*03:01	A*03:01	CASSLSNGYEQYF	V11-02	J02-07	CASSLSNxYEQYF+V11-02
A*03:01	A*03:01	CASSLSNHYEQYF	V11-02	J02-07	CASSLSNxYEQYF+V11-02
A*03:01	A*03:01	CASSLSNQYEQYF	V11-02	J02-07	CASSLSNxYEQYF+V11-02
A*03:01	A*03:01	CASSLSNSYEQYF	V11-02	J02-07	CASSLSNxYEQYF+V11-02
A*03:01	A*03:01	CASSAGQGARGYTF	V05-06	J01-02	CASSxGQGAxGYTF+V05-06
A*03:01	A*03:01	CASSRGQGASGYTF	V05-06	J01-02	CASSxGQGAxGYTF+V05-06
A*03:01	A*03:01	CASSSGQGAGGYTF	V05-06	J01-02	CASSxGQGAxGYTF+V05-06
A*03:01	A*03:01	CASSSGQGARGYTF	V05-06	J01-02	CASSxGQGAxGYTF+V05-06
A*03:01	A*03:01	CASSEHGGEQYF	V05-06	J02-07	CASSxHGxExxF+V05-06
A*03:01	A*03:01	CASSFHGAEQYF	V05-06	J02-07	CASSxHGxExxF+V05-06
A*03:01	A*03:01	CASSFHGDEQYF	V05-06	J02-07	CASSxHGxExxF+V05-06
A*03:01	A*03:01	CASSFHGGELFF	V05-06	J02-02	CASSxHGxExxF+V05-06
A*03:01	A*03:01	CASSFHGGEQFF	V05-06	J02-01	CASSxHGxExxF+V05-06
A*03:01	A*03:01	CASSFHGGEQYF	V05-06	J02-07	CASSxHGxExxF+V05-06
A*03:01	A*03:01	CASSFHGSEQYF	V05-06	J02-07	CASSxHGxExxF+V05-06
A*03:01	A*03:01	CASSGHGGEQYF	V05-06	J02-07	CASSxHGxExxF+V05-06
A*03:01	A*03:01	CASSIHGGEQFF	V05-06	J02-01	CASSxHGxExxF+V05-06
A*03:01	A*03:01	CASSIHGGEQYF	V05-06	J02-07	CASSxHGxExxF+V05-06
A*03:01	A*03:01	CASSLHGDEQYF	V05-06	J02-07	CASSxHGxExxF+V05-06
A*03:01	A*03:01	CASSLHGGEAFF	V05-06	J01-01	CASSxHGxExxF+V05-06
A*03:01	A*03:01	CASSLHGGELFF	V05-06	J02-02	CASSxHGxExxF+V05-06
A*03:01	A*03:01	CASSLHGGEQFF	V05-06	J02-01	CASSxHGxExxF+V05-06
A*03:01	A*03:01	CASSLHGGEQYF	V05-06	J02-07	CASSxHGxExxF+V05-06
A*03:01	A*03:01	CASSLHGNEQFF	V05-06	J02-01	CASSxHGxExxF+V05-06
A*03:01	A*03:01	CASSLHGNEQYF	V05-06	J02-07	CASSxHGxExxF+V05-06
A*03:01	A*03:01	CASSLHGSEQYF	V05-06	J02-07	CASSxHGxExxF+V05-06
A*03:01	A*03:01	CASSMHGGEQFF	V05-06	J02-01	CASSxHGxExxF+V05-06
A*03:01	A*03:01	CASSNHGGELFF	V05-06	J02-02	CASSxHGxExxF+V05-06
A*03:01	A*03:01	CASSNHGGEQYF	V05-06	J02-07	CASSxHGxExxF+V05-06
A*03:01	A*03:01	CASSPHGGELFF	V05-06	J02-02	CASSxHGxExxF+V05-06
A*03:01	A*03:01	CASSPHGGEQYF	V05-06	J02-07	CASSxHGxExxF+V05-06
A*03:01	A*03:01	CASSQHGGEQYF	V05-06	J02-07	CASSxHGxExxF+V05-06
A*03:01	A*03:01	CASSRHGGEQYF	V05-06	J02-07	CASSxHGxExxF+V05-06
A*03:01	A*03:01	CASSSHGDEQYF	V05-06	J02-07	CASSxHGxExxF+V05-06
A*03:01	A*03:01	CASSSHGGEAFF	V05-06	J01-01	CASSxHGxExxF+V05-06
A*03:01	A*03:01	CASSSHGGELFF	V05-06	J02-02	CASSxHGxExxF+V05-06
A*03:01	A*03:01	CASSSHGGEQFF	V05-06	J02-01	CASSxHGxExxF+V05-06
A*03:01	A*03:01	CASSSHGGEQYF	V05-06	J02-07	CASSxHGxExxF+V05-06
A*03:01	A*03:01	CASSSHGNEQYF	V05-06	J02-07	CASSxHGxExxF+V05-06
A*03:01	A*03:01	CASSTHGGEAFF	V05-06	J01-01	CASSxHGxExxF+V05-06
A*03:01	A*03:01	CASSTHGGEQFF	V05-06	J02-01	CASSxHGxExxF+V05-06
A*03:01	A*03:01	CASSTHGGEQYF	V05-06	J02-07	CASSxHGxExxF+V05-06
A*03:01	A*03:01	CASSVHGGEQYF	V05-06	J02-07	CASSxHGxExxF+V05-06
A*03:01	A*03:01	CASSYHGGEQYF	V05-06	J02-07	CASSxHGxExxF+V05-06
A*03:01	A*03:01	CASSEHGQPQHF	V05-06	J01-05	CASSxHGxPQHF+V05-06
A*03:01	A*03:01	CASSFHGGPQHF	V05-06	J01-05	CASSxHGxPQHF+V05-06
A*03:01	A*03:01	CASSFHGQPQHF	V05-06	J01-05	CASSxHGxPQHF+V05-06
A*03:01	A*03:01	CASSLHGGPQHF	V05-06	J01-05	CASSxHGxPQHF+V05-06
A*03:01	A*03:01	CASSLHGQPQHF	V05-06	J01-05	CASSxHGxPQHF+V05-06
A*03:01	A*03:01	CASSNHGGPQHF	V05-06	J01-05	CASSxHGxPQHF+V05-06
A*03:01	A*03:01	CASSPHGGPQHF	V05-06	J01-05	CASSxHGxPQHF+V05-06
A*03:01	A*03:01	CASSSHGGPQHF	V05-06	J01-05	CASSxHGxPQHF+V05-06
A*03:01	A*03:01	CASNPGQGLNEQFF	V09-01	J02-01	
A*03:01	A*03:01	CASSFRDLAYEQYF	V05-06	J02-07	
A*03:01	A*03:01	CASSLDRDTGELFF	V09-01	J02-02	
A*03:01	A*03:01	CASSLGTNSGNTIYF	V13-01	J01-03	

A*03:01	A*03:01	CASSLNRGREQYF	V12-03/12-04	J02-07	
A*03:01	A*03:01	CASSPKNTEAFF	V13-01	J01-01	
A*03:01	A*03:01	CASSPRQGPSTDTQYF	V27-01	J02-03	
A*03:01	A*03:01	CASSSDRGREKLFF	V13-01	J01-04	
C*02:02	C*02:02	CASSLSTYNEQFF	V05-06	J02-01	
DRB1*01:01	DRB1*01:01	CASSLDPGSETQYF	V05-06	J02-05	
DRB1*15:01	DRB1*15:01	CASSLDLGTDTQYF	V05-06	J02-03	CASSLDLxTDTQYF+V05-06
DRB1*15:01	DRB1*15:01	CASSLDLNTDTQYF	V05-06	J02-03	CASSLDLxTDTQYF+V05-06
DRB1*15:01	DRB1*15:01	CASSLDLRTDTQYF	V05-06	J02-03	CASSLDLxTDTQYF+V05-06
DRB1*15:01	DRB1*15:01	CASSLDLSTDTQYF	V05-06	J02-03	CASSLDLxTDTQYF+V05-06
DRB1*15:01	DRB1*15:01	CASSLDLTTDTQYF	V05-06	J02-03	CASSLDLxTDTQYF+V05-06
DRB1*15:01	DRB1*15:01	CASSPAPADEKLFF	V07-02	J01-04	CASSPAxADEKLFF+V07-02
DRB1*15:01	DRB1*15:01	CASSPARADEKLFF	V07-02	J01-04	CASSPAxADEKLFF+V07-02
DRB1*15:01	DRB1*15:01	CASSYSGSHFTDTQYF	V06-05	J02-03	CASSYSGSHxTDTQYF+V06-05
DRB1*15:01	DRB1*15:01	CASSYSGSHHTDTQYF	V06-05	J02-03	CASSYSGSHxTDTQYF+V06-05
DRB1*15:01	DRB1*15:01	CASSYSGSHPTDTQYF	V06-05	J02-03	CASSYSGSHxTDTQYF+V06-05
DRB1*15:01	DRB1*15:01	CASSYSGSHRTDTQYF	V06-05	J02-03	CASSYSGSHxTDTQYF+V06-05
DRB1*15:01	DRB1*15:01	CASSYSGSHSTDTQYF	V06-05	J02-03	CASSYSGSHxTDTQYF+V06-05
DRB1*15:01	DRB1*15:01	CASSYSGSHYTDTQYF	V06-05	J02-03	CASSYSGSHxTDTQYF+V06-05
DRB1*15:01	DRB1*15:01	CASSLAGGLDQPQHF	V05-06	J01-05	CASSxAGGLDQPQHF+V05-06
DRB1*15:01	DRB1*15:01	CASSSAGGLDQPQHF	V05-06	J01-05	CASSxAGGLDQPQHF+V05-06
DRB1*15:01	DRB1*15:01	CASSPGGAGQPQHF	V05-06	J01-05	CASSxGGAGQPQHF+V05-06
DRB1*15:01	DRB1*15:01	CASSSGGAGQPQHF	V05-06	J01-05	CASSxGGAGQPQHF+V05-06
DRB1*15:01	DRB1*15:01	CASSFSVRGGETQYF	V12-X	J02-05	CASSxSVRxxETQYF+V12-X
DRB1*15:01	DRB1*15:01	CASSLSVRAGETQYF	V12-X	J02-05	CASSxSVRxxETQYF+V12-X
DRB1*15:01	DRB1*15:01	CASSLSVRGAETQYF	V12-X	J02-05	CASSxSVRxxETQYF+V12-X
DRB1*15:01	DRB1*15:01	CASSLSVRGGETQYF	V12-X	J02-05	CASSxSVRxxETQYF+V12-X
DRB1*15:01	DRB1*15:01	CASSLSVRGLETQYF	V12-X	J02-05	CASSxSVRxxETQYF+V12-X
DRB1*15:01	DRB1*15:01	CASSLSVRGQETQYF	V12-X	J02-05	CASSxSVRxxETQYF+V12-X
DRB1*15:01	DRB1*15:01	CASSFSVSGANVLTF	V12-X	J02-06	CASSxSVSGANVLTF+V12-X
DRB1*15:01	DRB1*15:01	CASSLSVSGANVLTF	V12-X	J02-06	CASSxSVSGANVLTF+V12-X
DRB1*15:01	DRB1*15:01	CASSRSVSGANVLTF	V12-X	J02-06	CASSxSVSGANVLTF+V12-X
DRB1*15:01	DRB1*15:01	CASRTGGGPSGNTIYF	V28-01	J01-03	
DRB1*15:01	DRB1*15:01	CASSEGRLYGYTF	V28-01	J01-02	
DRB1*15:01	DRB1*15:01	CASSFSGQGEKLFF	V12-X	J01-04	
DRB1*15:01	DRB1*15:01	CASSGPMNTEAFF	V25-01	J01-01	
DRB1*15:01	DRB1*15:01	CASSLAGGLDSPLHF	V05-06	J01-06	
DRB1*15:01	DRB1*15:01	CASSLGRTSTDTQYF	V13-01	J02-03	
DRB1*15:01	DRB1*15:01	CASSPTGFGYTF	V05-06	J01-02	
DRB1*15:01	DRB1*15:01	CASSSDPYGYTF	V28-01	J01-02	
DRB1*15:01	DRB1*15:01	CASSWTGTNEKLFF	V05-06	J01-04	
DRB5*02:02	DRB5*02:02	CASSQTGNSPLHF	V04-03	J01-06	
uncertain	A*03:01	CASSVEGLARNEQFF	V09-01	J02-01	
uncertain	DRB1*04:01	CAWGGGYGYTF	V30-01	J01-02	

Table S1: HLA ($p < 0.0001$): the HLA allele suggested by a FET p-value threshold of 0.0001. In case no association was below this threshold, the association was labelled as "uncertain". HLA ($p < 0.001$): the HLA allele suggested by a FET p-value threshold of 0.001. CDR3beta: amino acid sequences of the complementary determining region 3 (CDR3) of the sequenced beta chain of the TCR. Vbeta: variable beta chain of the sequenced TCR. Jbeta: junctional beta chain of the sequenced TCR. Motif: common motif that groups several of the shared TCR sequences (CDR3 + Vbeta) into one cluster with a wildcard ("x") defined by a Hamming distance of <=1.

Table S2: T-cell receptor alpha and beta chain data from single-cell RNA sequencing validation
Given are the 10x Cellranger VDJ outputs in one table with the following column names added: "file_name" refers to the respective patient sample, "pattern_match" indicating whether the T-cell receptor matches a sequence from Supplementary Table 1, and "barcode_unique" which is a concatenated string of barcode and patient sample to allow for analysis over several patient samples at once, even if a barcode might be present in more than one sample.

Table S3: Model outputs

Given are the modelling outputs used for the statistical assessment of the clinical outcome parameters of Fig. 2 and Fig. S2-5. The sheets are named according to the respective figure panel and whether it is a Schoenfeld residual output, or the dataset was restricted to HLA-A*03(:01) positive or -negative patients, or older or younger patients.

01: Figure panel 2b model output
02: Figure panel 2b Schoenfeld residuals
03: Figure panel 2b model output restricted to A*03 negative patients
04: Figure panel 2b model output restricted to A*03 positive patients
05: Figure panel 2d model output
06: Figure panel 2d Schoenfeld residuals
07: Figure panel 2d model output restricted to younger patients
08: Figure panel 2d model output restricted to older patients
09: Figure panel 2d model output restricted to A*03 negative patients
10: Figure panel 2d model output restricted to A*03 positive patients
11: Figure panel 2f model output
12: Figure panel 2f model output restricted to A*03 negative patients
13: Figure panel 2f model output restricted to A*03 positive patients
14: Figure panel 2j model output
15: Figure panel 2j model output restricted to A*03 negative patients
16: Figure panel 2j model output restricted to A*03 positive patients
17: Figure panel 2h model output
18: Figure panel 2h model output restricted to A*03 negative patients
19: Figure panel 2h model output restricted to A*03 positive patients
20: Figure panel S2b model output
21: Figure panel S2b Schoenfeld residuals
22: Figure panel S2b model output restricted to A*03 negative patients
23: Figure panel S2b model output restricted to A*03 positive patients
24: Figure panel S2d model output
25: Figure panel S2d model output restricted to A*03 negative patients
26: Figure panel S2d model output restricted to A*03 positive patients
27: Figure panel S3b model output
28: Figure panel S3b model output restricted to A*03 negative patients
29: Figure panel S3b model output restricted to A*03 positive patients
30: Figure panel S3d model output
31: Figure panel S3d model output restricted to A*03 negative patients
32: Figure panel S3d model output restricted to A*03 positive patients
33: Figure panel S3f model output
34: Figure panel S3f model output restricted to A*03 negative patients
35: Figure panel S3f model output restricted to A*03 positive patients
36: Figure panel S3h model output
37: Figure panel S3h model output restricted to A*03 negative patients
38: Figure panel S3h model output restricted to A*03 positive patients
39: Figure panel S4b model output
40: Figure panel S4b model output restricted to A*03 negative patients
41: Figure panel S4b model output restricted to A*03 positive patients
42: Figure panel S4d model output
43: Figure panel S4d model output restricted to A*03 negative patients
44: Figure panel S4b model output restricted to A*03 positive patients
45: Figure panel S4f model output
46: Figure panel S4f model output restricted to A*03 negative patients
47: Figure panel S4f model output restricted to A*03 positive patients
48: Figure panel S5b model output
49: Figure panel S5b model output restricted to A*03 negative patients
50: Figure panel S5b model output restricted to A*03 positive patients
51: Figure panel S5d model output

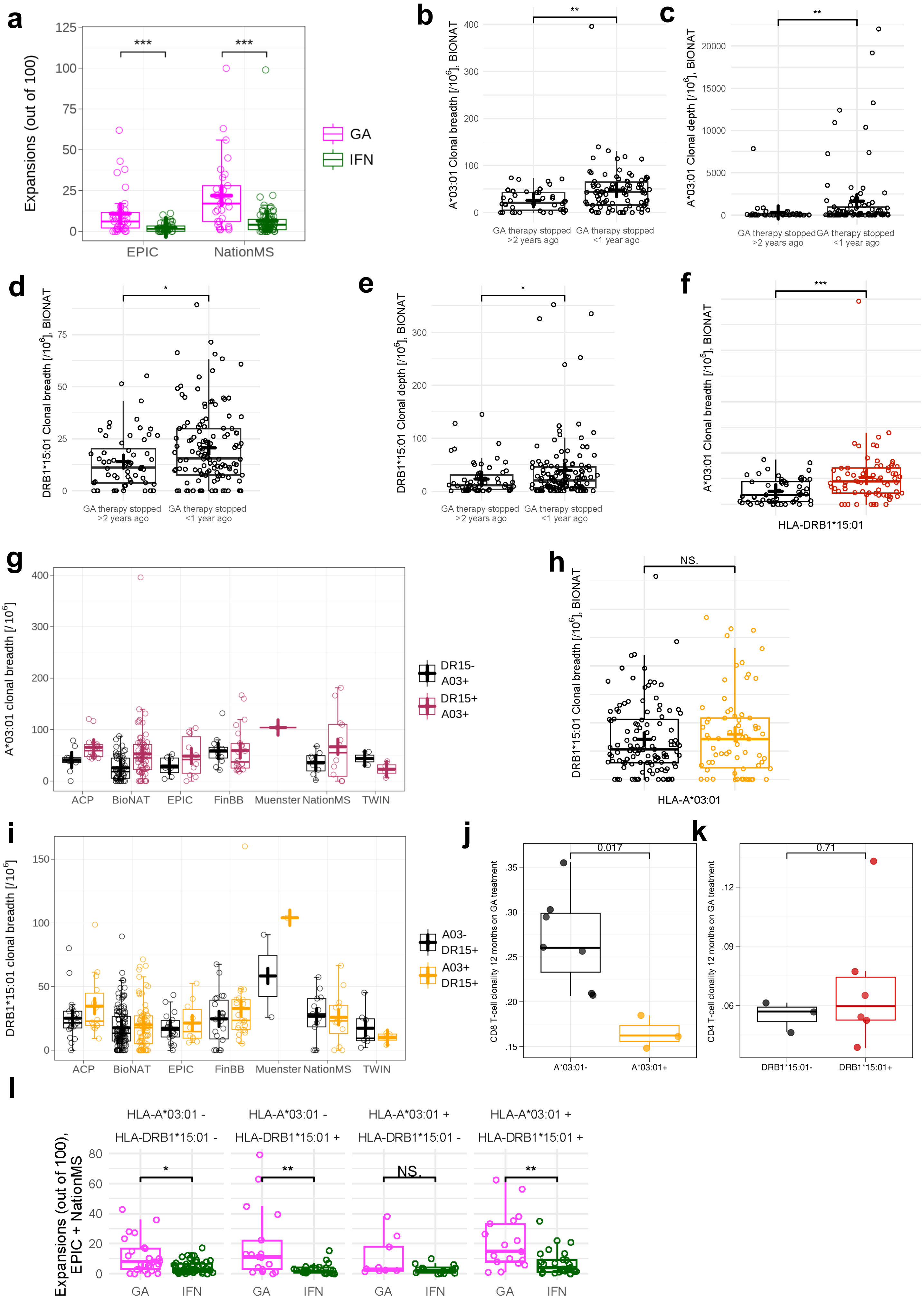
a
Expansions (out of 100)
125
100
75
50
25
0
***
***
EPIC
NationMS
GA
IFN
b
A*03:01 Clonal breadth [/10^6], BIONAT
**
400
300
200
100
0
GA therapy stopped >2 years ago
GA therapy stopped <1 year ago
c
A*03:01 Clonal depth [/10^6], BIONAT
**
20000
15000
10000
5000
0
GA therapy stopped >2 years ago
GA therapy stopped <1 year ago
d
DRB1*15:01 Clonal breadth [/10^6], BIONAT
*
75
50
25
0
GA therapy stopped >2 years ago
GA therapy stopped <1 year ago
e
DRB1*15:01 Clonal depth [/10^6], BIONAT
*
300
200
100
0
GA therapy stopped >2 years ago
GA therapy stopped <1 year ago
f
A*03:01 Clonal breadth [/10^6], BIONAT
***
HLA-DRB1*15:01
g
A*03:01 clonal breadth [/10^6]
400
300
200
100
0
ACP
BioNAT
EPIC
FinBB
Muenster
NationMS
TWIN
DR15- A03+
DR15+ A03+
h
DRB1*15:01 Clonal breadth [/10^6], BIONAT
NS.
HLA-A*03:01
i
DRB1*15:01 clonal breadth [/10^6]
150
100
50
0
ACP
BioNAT
EPIC
FinBB
Muenster
NationMS
TWIN
A03- DR15+
A03+ DR15+
j
CD8 T-cell clonality 12 months on GA treatment
0.017
.35
.30
.25
.20
.15
A*03:01-
A*03:01+
k
CD4 T-cell clonality 12 months on GA treatment
0.71
.12
.09
.06
DRB1*15:01-
DRB1*15:01+
l
Expansions (out of 100), EPIC + NationMS
HLA-A*03:01 - HLA-DRB1*15:01 -
HLA-A*03:01 - HLA-DRB1*15:01 +
HLA-A*03:01 + HLA-DRB1*15:01 -
HLA-A*03:01 + HLA-DRB1*15:01 +
80
60
40
20
0
*
**
NS.
**
GA
IFN

Figure S1: Quantification of GA-associated sequences and expansions

a: Number of expanded T-cell clones out of a maximum of 100 in pre- vs. on-treatment samples from the EPIC and NationMS cohorts (EPIC: n(GA)=35, n(IFN)=32, p=0.00051; NationMS: n(GA)=29, n(IFN)=68, p<0.0001). Magenta indicates GA treatment, green indicates IFN treatment.
b and c: Assessment of the HLA-A*03:01 GA-associated sequence pattern clonal breadth (b, p=0.004) and depth (c, p=0.0022) in HLA-A*03:01 carrying patients with MS of the BIONAT cohort, either more than two years after GA treatment (left column, n=38) or after less than one year after GA treatment (right column, n=86).
d and e: Assessment of the HLA-DRB1*15:01 GA-associated sequence pattern clonal breadth (d, p=0.022) and depth (e, p=0.047) in HLA-DRB1*15:01 carrying patients with MS of the BIONAT cohort, either more than two years after GA treatment (left column, n=60) or after less than one year after GA treatment (right column, n=126).
f and g: Assessment of the HLA-A*03:01 GA-associated sequence pattern clonal breadth in HLA-A*03:01 carrying patients carrying (red, n=70) or not carrying (black, n=56) the HLA-DRB1*15:01 allele, in BIONAT (f, p=0.00013) or split by cohorts (g). Number of patients: HLA-A*03:01 positive HLA-DRB1*15:01 negative: ACP: 9, BIONAT: 56, EPIC: 7, FinBB: 12, NationMS: 8, MS Twin: 2; HLA-A*03:01 positive HLA-DRB1*15:01 positive: ACP: 14, BIONAT: 70, EPIC: 10, FinBB: 22, Muenster: 1, NationMS: 12, MS Twin: 4.
h and i: Assessment of the HLA-DRB1*15:01 GA-associated sequence pattern clonal breadth in HLA-DRB1*15:01 carrying patients carrying (yellow, n=70) or not carrying (black, n=118) the HLA-A*03:01 allele, in BIONAT (h, p=0.61) or split by cohorts (i). Number of patients: HLA-DRB1*15:01 positive HLA-A*03:01 negative: ACP: 21, BIONAT: 118, EPIC: 18, FinBB: 22, Muenster: 2, NationMS: 13, MS Twin: 7; HLA-DRB1*15:01 positive HLA-A*03:01 positive: ACP: 14, BIONAT: 70, EPIC: 10, FinBB: 22, Muenster: 1, NationMS: 12, MS Twin: 4.
j and k: Clonality of sorted CD8+ (j, n=10) and CD4+ T cells (k, n=9) of patients at twelve months of GA treatment.
l: Number of expanded T-cell clones out of a maximum of 100 in pre- vs. on-treatment samples from the EPIC and NationMS cohorts stratified by HLA-A*03:01 and HLA-DRB1*15:01 status (HLA-A*03:01 negative HLA-DRB1*15:01 negative: n(GA)=25, n(IFN)=40, p=0.029; HLA-A*03:01 negative HLA-DRB1*15:01 positive: n(GA)=15, n(IFN)=23, p=0.0079; HLA-A*03:01 positive HLA-DRB1*15:01 negative: n(GA)=8, n(IFN)=15, p=0.2; HLA-A*03:01 positive HLA-DRB1*15:01 positive: n(GA)=16, n(IFN)=22, p=0.0017).

As shorthand, A03 and DR15 are used to refer to HLA-A*03:01 and HLA-DRB1*15:01. Boxes indicate the 25% and 75% percentile and median, whiskers indicate 1.5x inter-quartile range, + indicates the mean. Significance was assessed by a Wilcoxon rank-sum test. The asterisks indicate a p-value of <0.05 (*), <0.01 (**), or <0.001(***).

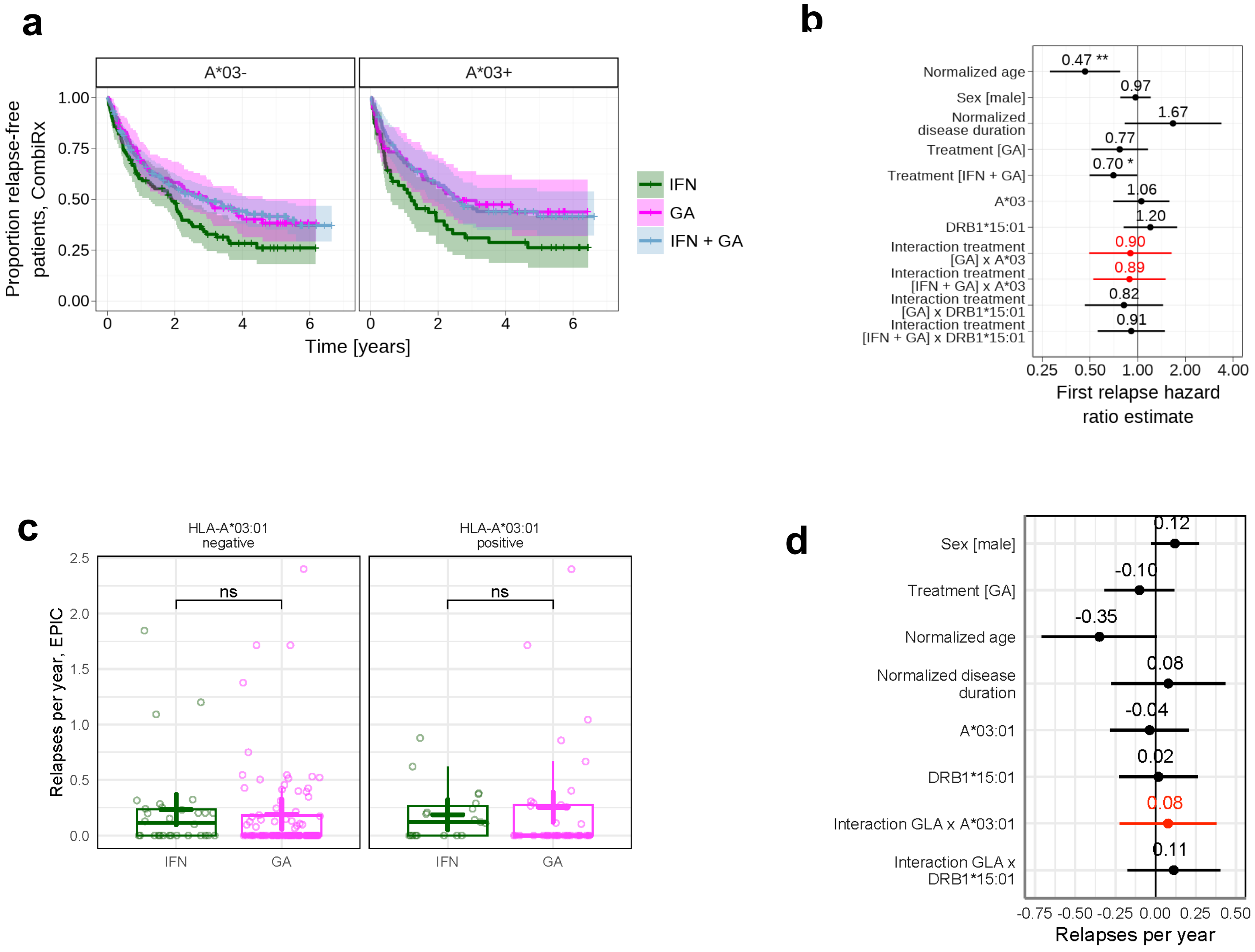
a
A*03-
A*03+
Proportion relapse-free patients, CombiRx
1.00
0.75
0.50
0.25
0.00
0
2
4
6
Time [years]
IFN
GA
IFN + GA
b
Normalized age
0.47 **
Sex [male]
0.97
Normalized disease duration
1.67
Treatment [GA]
0.77
Treatment [IFN + GA]
0.70 *
A*03
1.06
DRB1*15:01
1.20
Interaction treatment [GA] x A*03
0.90
Interaction treatment [IFN + GA] x A*03
0.89
Interaction treatment [GA] x DRB1*15:01
0.82
Interaction treatment [IFN + GA] x DRB1*15:01
0.91
0.25
0.50
1.00
2.00
4.00
First relapse hazard ratio estimate
c
HLA-A*03:01 negative
HLA-A*03:01 positive
Relapses per year, EPIC
2.5
2.0
1.5
1.0
0.5
0.0
ns
IFN
GA
d
Sex [male]
0.12
Treatment [GA]
-0.10
Normalized age
-0.35
Normalized disease duration
0.08
A*03:01
-0.04
DRB1*15:01
0.02
Interaction GLA x A*03:01
0.08
Interaction GLA x DRB1*15:01
0.11
-0.75
-0.50
-0.25
0.00
0.25
0.50
Relapses per year

Figure S2: Relapse data

a: Kaplan-Meier curves with proportions of relapse-free patients (y-axis) vs observation period in years (x-axis) in HLA-A*03-negative (left panel, n(IFN)=86, n(GA)=97, n(IFN + GA)=206) and HLA-A*03-positive patients (right panel, n(IFN)=44, n(GA)=50, n(IFN + GA)=80) of the CombiRx cohort.
b: Cox regression model of panel a for proportions of relapse-free patients of the CombiRx cohort with the covariates normalised age, sex, normalised disease duration, GA treatment, GA + IFN treatment, HLA-A*03, HLA-DRB1*15:01, and the interactions of treatment and HLA covariates yields a p-value of 0.7295 for the HLA-A*03 GA interaction and a p-value of 0.6575 for the HLA-A*03 IFN + GA interaction.
c: Relapses per year in patients of the EPIC cohort (HLA-A*03:01 negative: n(IFN)=26, n(GA)=57, p=0.28; HLA-A*03:01 positive: n(IFN)=19, n(GA)=23, p=0.42).
d: Modelling the data from panel c with a linear regression and the covariates normalised age, sex, normalised disease duration, treatment, HLA-A*03:01, HLA-DRB1*15:01, and the interactions of treatment and HLA covariates yields a p-value of 0.3764 for the HLA-A*03:01 GA interaction.
Green bars and lines indicate IFN treatment, magenta bars and lines GA treatment and light blue IFN + GA treatment; Boxes indicate the 25% and 75% percentile and median, whiskers indicate 1.5x inter-quartile range, + indicates the mean. As shorthand, A*03, A*03:01 and DRB1*15:01 are used to refer to HLA-A*03, HLA-A*03:01 and HLA-DRB1*15:01.

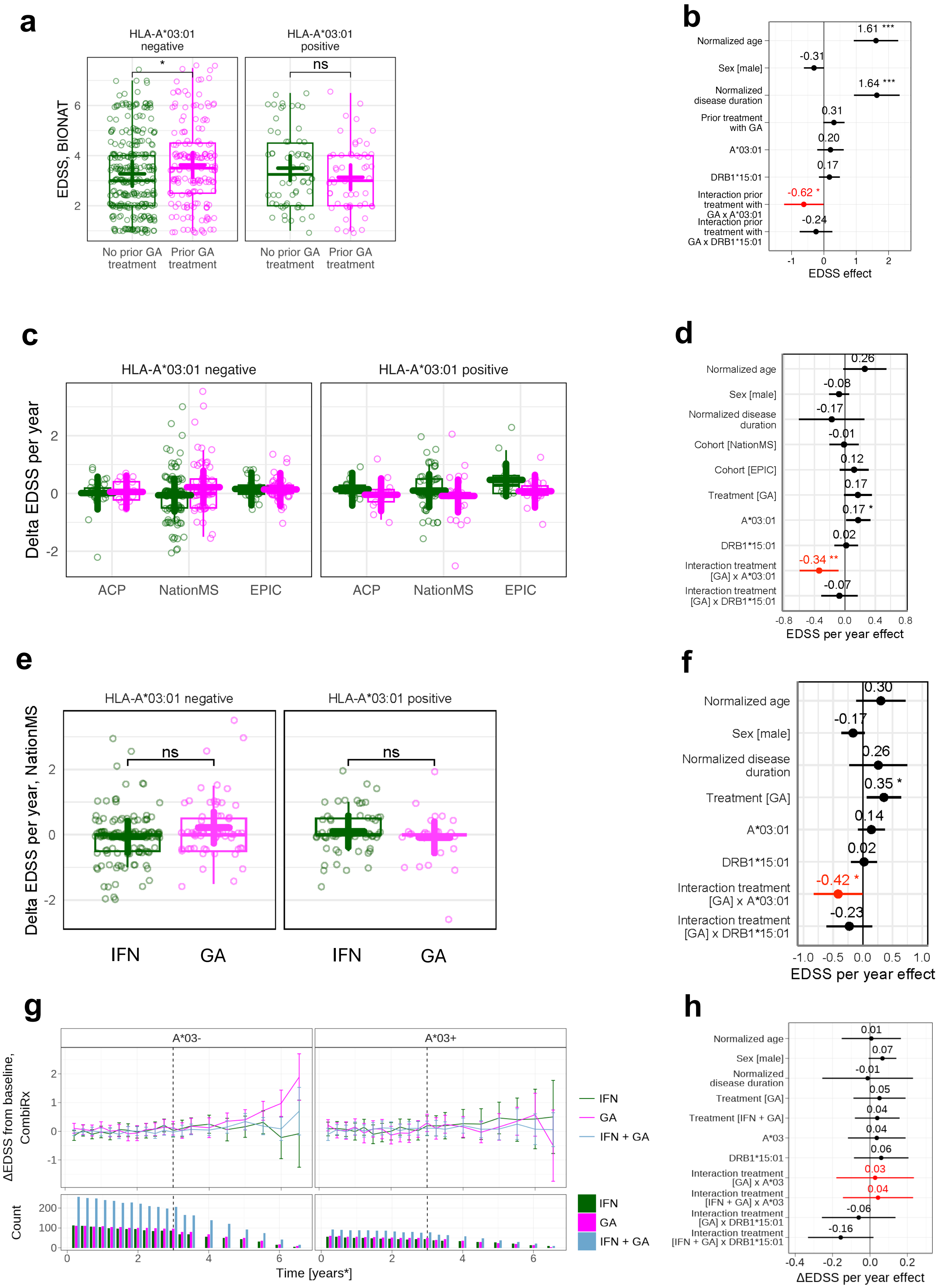

a
HLA-A*03:01 negative
HLA-A*03:01 positive
EDSS, BIONAT
*
ns
No prior GA treatment
Prior GA treatment
b
Normalized age
Sex [male]
Normalized disease duration
Prior treatment with GA
A*03:01
DRB1*15:01
Interaction prior treatment with GA x A*03:01
Interaction prior treatment with GA x DRB1*15:01
1.61 ***
-0.31
1.64 ***
0.31
0.20
0.17
-0.62 *
-0.24
EDSS effect
c
HLA-A*03:01 negative
HLA-A*03:01 positive
Delta EDSS per year
ACP
NationMS
EPIC
d
Normalized age
Sex [male]
Normalized disease duration
Cohort [NationMS]
Cohort [EPIC]
Treatment [GA]
A*03:01
DRB1*15:01
Interaction treatment [GA] x A*03:01
Interaction treatment [GA] x DRB1*15:01
0.26
-0.08
-0.17
-0.01
0.12
0.17
0.17 *
0.02
-0.34 **
-0.07
EDSS per year effect
e
HLA-A*03:01 negative
HLA-A*03:01 positive
Delta EDSS per year, NationMS
ns
IFN
GA
f
Normalized age
Sex [male]
Normalized disease duration
Treatment [GA]
A*03:01
DRB1*15:01
Interaction treatment [GA] x A*03:01
Interaction treatment [GA] x DRB1*15:01
0.30
-0.17
0.26
0.35 *
0.14
0.02
-0.42 *
-0.23
EDSS per year effect
g
A*03-
A*03+
ΔEDSS from baseline, CombiRx
Count
IFN
GA
IFN + GA
Time [years*]
h
Normalized age
Sex [male]
Normalized disease duration
Treatment [GA]
Treatment [IFN + GA]
A*03
DRB1*15:01
Interaction treatment [GA] x A*03
Interaction treatment [IFN + GA] x A*03
Interaction treatment [GA] x DRB1*15:01
Interaction treatment [IFN + GA] x DRB1*15:01
0.01
0.07
-0.01
0.05
0.04
0.04
0.06
0.03
0.04
-0.06
-0.16
ΔEDSS per year effect

Figure S3: EDSS data

a: EDSS scores of matched patients from the BIONAT cohort without prior GA treatment (green; n(HLA-A*03:01 negative)=301, n(HLA-A*03:01 positive)=65, p=0.018) and with prior GA treatment (magenta; n(HLA-A*03:01 negative)=178, n(HLA-A*03:01 positive)=56, p=0.26).
b: Linear model of panel a for EDSS scores of the BIONAT cohort with the covariates normalised age, sex, normalised disease duration, prior GA treatment, HLA-A*03:01 status, HLA-DRB1*15:01 status, and the interactions of treatment and HLA covariates yields a p-value of 0.0455 for the HLA-A*03:01 GA interaction.
c: Annual change in EDSS scores of ACP, NationMS and EPIC cohorts; Left panel indicates HLA-A*03:01 negative patients (ACP: n(IFN)=17, n(GA)=14; NationMS: n(IFN)=140, n(GA)=60; EPIC: n(IFN)=24, n(GA)=55), right panel HLA-A*03:01 positive patients (ACP: n(IFN)=11, n(GA)=12; NationMS: n(IFN)=70, n(GA)=29; EPIC: n(IFN)=17, n(GA)=21).
d: Linear model of panel c for annual change in EDSS scores with the covariates normalised age, sex, normalised disease duration, cohort (NationMS, EPIC, ACP), GA treatment, HLA-A*03:01 status, HLA-DRB1*15:01 status, and the interactions of treatment and HLA covariates yields a p-value of 0.0085 for the HLA-A*03:01 GA interaction.
e: Annual change in EDSS scores of the NationMS cohort; Left panel indicates HLA-A*03:01 non-carrying patients (n(IFN)=140, n(GA)=60, p=0.13), right panel HLA-A*03:01 carrying patients (n(IFN)=70, n(GA)=29, p=0.3).
f: Linear model of panel e for annual change in EDSS scores with the covariates normalised age, sex, normalised disease duration, GA treatment, HLA-A*03:01 status, HLA-DRB1*15:01 status, and the interactions of treatment and HLA covariates yields a p-value of 0.0459 for the HLA-A*03:01 GA interaction.
g: Change in EDSS scores from baseline with 95% confidence interval vs observation time of the CombiRx cohort; Left panel indicates HLA-A*03 non-carrying patients (n(IFN)=86, n(GA)=97, n(IFN + GA)=206), right panel indicates HLA-A*03 carrying patients (n(IFN)=44, n(GA)=50, n(IFN + GA)=80). Count indicates number of patients with a measurement at that time point. The dashed line indicates the three-year mark for analysis.
h: Linear model of panel g for annual changes in EDSS scores with the covariates normalised age, sex, normalised disease duration, GA treatment, GA + IFN treatment, HLA-A*03 status, HLA-DRB1*15:01 status, and the interactions of treatment and HLA covariates yields a p-value of 0.7940 for the HLA-A*03 GA interaction and a p-value of 0.6524 for the HLA-A*03 IFN + GA interaction.
Green bars and lines indicate IFN treatment, magenta bars and lines GA treatment and light blue IFN + GA treatment; Boxes indicate the 25% and 75% percentile and median, whiskers indicate 1.5x inter-quartile range, + indicates the mean. As shorthand, A*03, A*03:01 and DRB1*15:01 are used to refer to HLA-A*03, HLA-A*03:01 and HLA-DRB1*15:01.

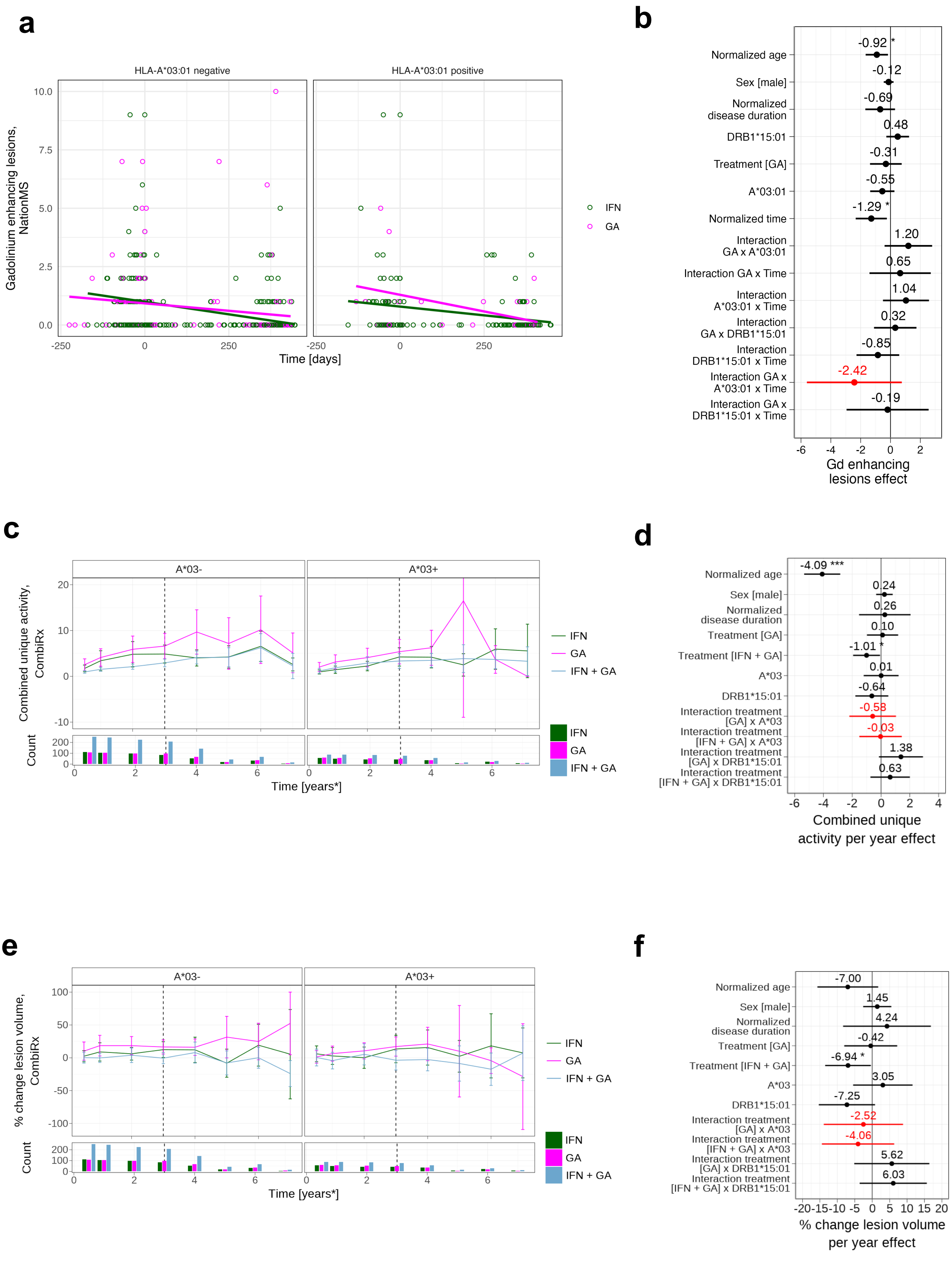

a
HLA-A*03:01 negative
HLA-A*03:01 positive
Gadolinium enhancing lesions, NationMS
10.0
7.5
5.0
2.5
0.0
-250
0
250
Time [days]
IFN
GA
b
Normalized age
Sex [male]
Normalized disease duration
DRB1*15:01
Treatment [GA]
A*03:01
Normalized time
Interaction GA x A*03:01
Interaction GA x Time
Interaction A*03:01 x Time
Interaction GA x DRB1*15:01
Interaction DRB1*15:01 x Time
Interaction GA x A*03:01 x Time
Interaction GA x DRB1*15:01 x Time
-0.92 *
-0.12
-0.69
0.48
-0.31
-0.55
-1.29 *
1.20
0.65
1.04
0.32
-0.85
-2.42
-0.19
Gd enhancing lesions effect
c
A*03-
A*03+
Combined unique activity, CombiRx
Count
Time [years*]
IFN
GA
IFN + GA
d
Normalized age
Sex [male]
Normalized disease duration
Treatment [GA]
Treatment [IFN + GA]
A*03
DRB1*15:01
Interaction treatment [GA] x A*03
Interaction treatment [IFN + GA] x A*03
Interaction treatment [GA] x DRB1*15:01
Interaction treatment [IFN + GA] x DRB1*15:01
-4.09 ***
0.24
0.26
0.10
-1.01 *
0.01
-0.64
-0.58
-0.03
1.38
0.63
Combined unique activity per year effect
e
A*03-
A*03+
% change lesion volume, CombiRx
Count
Time [years*]
IFN
GA
IFN + GA
f
Normalized age
Sex [male]
Normalized disease duration
Treatment [GA]
Treatment [IFN + GA]
A*03
DRB1*15:01
Interaction treatment [GA] x A*03
Interaction treatment [IFN + GA] x A*03
Interaction treatment [GA] x DRB1*15:01
Interaction treatment [IFN + GA] x DRB1*15:01
-7.00
1.45
4.24
-0.42
-6.94 *
3.05
-7.25
-2.52
-4.06
5.62
6.03
% change lesion volume per year effect

Figure S4: MRI data

a: Gadolinium enhancing lesions vs time in days of the NationMS cohort. Left panel indicates HLA-A*03:01 non-carrying patients (n(IFN)=173, n(GA)=65), right panel HLA-A*03:01 carrying patients (n(IFN)=78, n(GA)=26).
b: Linear mixed model of panel a for Gadolinium enhancing lesions of the NationMS cohort with the covariates normalised age, sex, normalised disease duration, normalised observation time, GA treatment, HLA-A*03:01 status, HLA-DRB1*15:01 status, and the interactions of treatment, observation time and HLA covariates yields a p-value of 0.1356 for the HLA-A*03:01 GA interaction.
c: Combined unique activity (CUA) with 95% confidence interval vs observation time in years of the CombiRx cohort; Left panel indicates HLA-A*03 non-carrying patients (n(IFN)=84, n(GA)=98, n(IFN + GA)=207), right panel indicates HLA-A*03 carrying patients (n(IFN)=43, n(GA)=50, n(IFN + GA)=76). Count indicates number of patients with a measurement at that time point. The dashed line indicates the three-year mark for analysis.
d: Linear model of panel c for combined unique activity per year of the CombiRx cohort with the covariates normalised age, sex, normalised disease duration, GA treatment, IFN + GA treatment, HLA-A*03 status, DRB1*15:01 status, and the interactions of treatment and HLA covariates yields a p-value of 0.4803 for the HLA-A*03 GA interaction and 0.9650 for the HLA-A*03 IFN + GA interaction.
e: Percent change in T1 and T2 lesion volume with 95% confidence interval vs observation time of the CombiRx cohort; Left panel indicates HLA-A*03 non-carrying patients (n(IFN)=84, n(GA)=98, n(IFN + GA)=207), right panel indicates HLA-A*03 carrying patients (n(IFN)=43, n(GA)=50, n(IFN + GA)=76). Count indicates number of patients with a measurement at that time point. The dashed line indicates the three-year mark for analysis.
f: Linear model of panel c for percent change in T1 and T2 lesion volume per year of the CombiRx cohort with the covariates normalised age, sex, normalised disease duration, GA treatment, IFN + GA treatment, HLA-A*03 status, DRB1*15:01 status and the interactions of treatment and HLA covariates yields a p-value of 0.6631 for the HLA-A*03 GA interaction and a p-value of 0.4418 for the HLA-A*03 IFN + GA interaction.
Green bars and lines indicate IFN treatment, magenta bars and lines GA treatment and light blue IFN + GA treatment; Boxes indicate the 25% and 75% percentile and median, whiskers indicate 1.5x inter-quartile range, + indicates the mean. As shorthand, A*03, A*03:01 and DRB1*15:01 are used to refer to HLA-A*03, HLA-A*03:01 and HLA-DRB1*15:01.

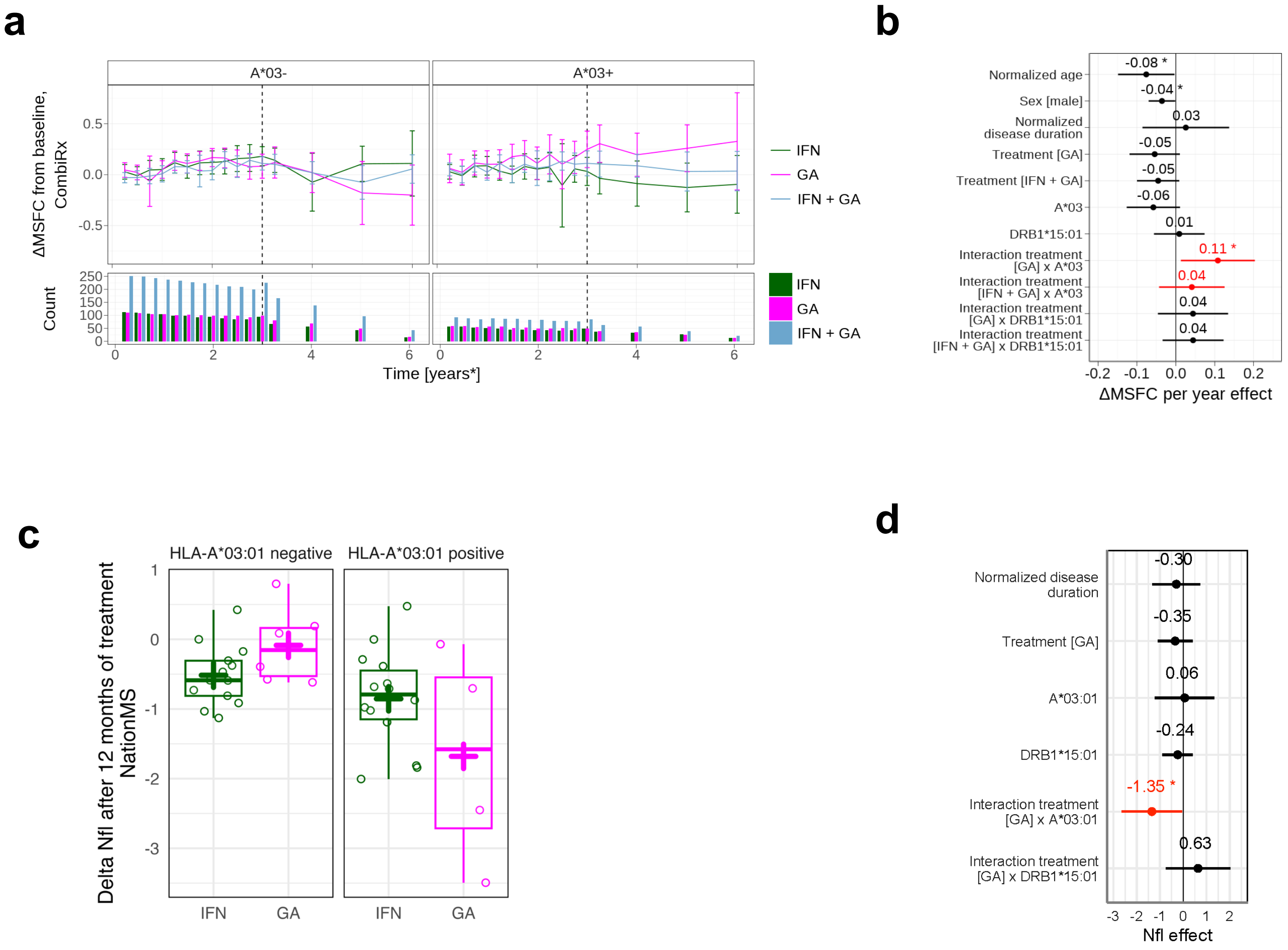
a
A*03-
A*03+
ΔMSFC from baseline, CombiRx
Count
Time [years*]
IFN
GA
IFN + GA
b
Normalized age
Sex [male]
Normalized disease duration
Treatment [GA]
Treatment [IFN + GA]
A*03
DRB1*15:01
Interaction treatment [GA] x A*03
Interaction treatment [IFN + GA] x A*03
Interaction treatment [GA] x DRB1*15:01
Interaction treatment [IFN + GA] x DRB1*15:01
-0.08 *
-0.04 *
0.03
-0.05
-0.05
-0.06
0.01
0.11 *
0.04
0.04
0.04
ΔMSFC per year effect
c
HLA-A*03:01 negative
HLA-A*03:01 positive
Delta Nfl after 12 months of treatment NationMS
IFN
GA
d
Normalized disease duration
Treatment [GA]
A*03:01
DRB1*15:01
Interaction treatment [GA] x A*03:01
Interaction treatment [GA] x DRB1*15:01
-0.30
-0.35
0.06
-0.24
-1.35 *
0.63
Nfl effect

Figure S5: Additional measures

a: Change in MSFC scores from baseline with 95% confidence interval vs observation time in the CombiRx cohort. Left panel indicates HLA-A*03-negative patients (n(IFN)=95, n(GA)=100, n(IFN + GA)=225), right panel indicates HLA-A*03-positive patients (n(IFN)=49, n(GA)=49, n(IFN + GA)=86). Count indicates number of patients with a measurement at that time point. The dashed line indicates the three-year mark for analysis.
b: Linear model of panel a for annual changes of MSFC in the CombiRx cohort with the covariates normalised age, sex, disease duration, GA treatment, IFN + GA treatment, HLA-A*03 status, DRB1*15:01 status, and the interactions of treatment and HLA covariates yields a p-value of 0.0260 for the HLA-A*03 GA interaction and a p-value of 0.3368 for the HLA-A*03 IFN + GA interaction.
c: Change in normalised Nfl values from baseline to one year after treatment in the NationMS cohort. Left panel indicates HLA-A*03:01 non-carrying patients (n(IFN)=15, n(GA)=6), right panel HLA-A*03:01 carrying patients (n(IFN)=14, n(GA)=5).
d: Linear model of panel c for change in normalised Nfl values after one year of treatment in the NationMS cohort with the covariates GA treatment, HLA-A*03:01 status, DRB1*15:01 status, and the interactions of treatment and HLA covariates yields a p-value of 0.0410 for the HLA-A*03:01 GA interaction.
Green bars and lines indicate IFN treatment, magenta bars and lines GA treatment and light blue IFN + GA treatment. Boxes indicate the 25% and 75% percentile and median, whiskers indicate 1.5x inter-quartile range, + indicates the mean. As shorthand, A*03, A*03:01 and DRB1*15:01 are used to refer to HLA-A*03, HLA-A*03:01 and HLA-DRB1*15:01.

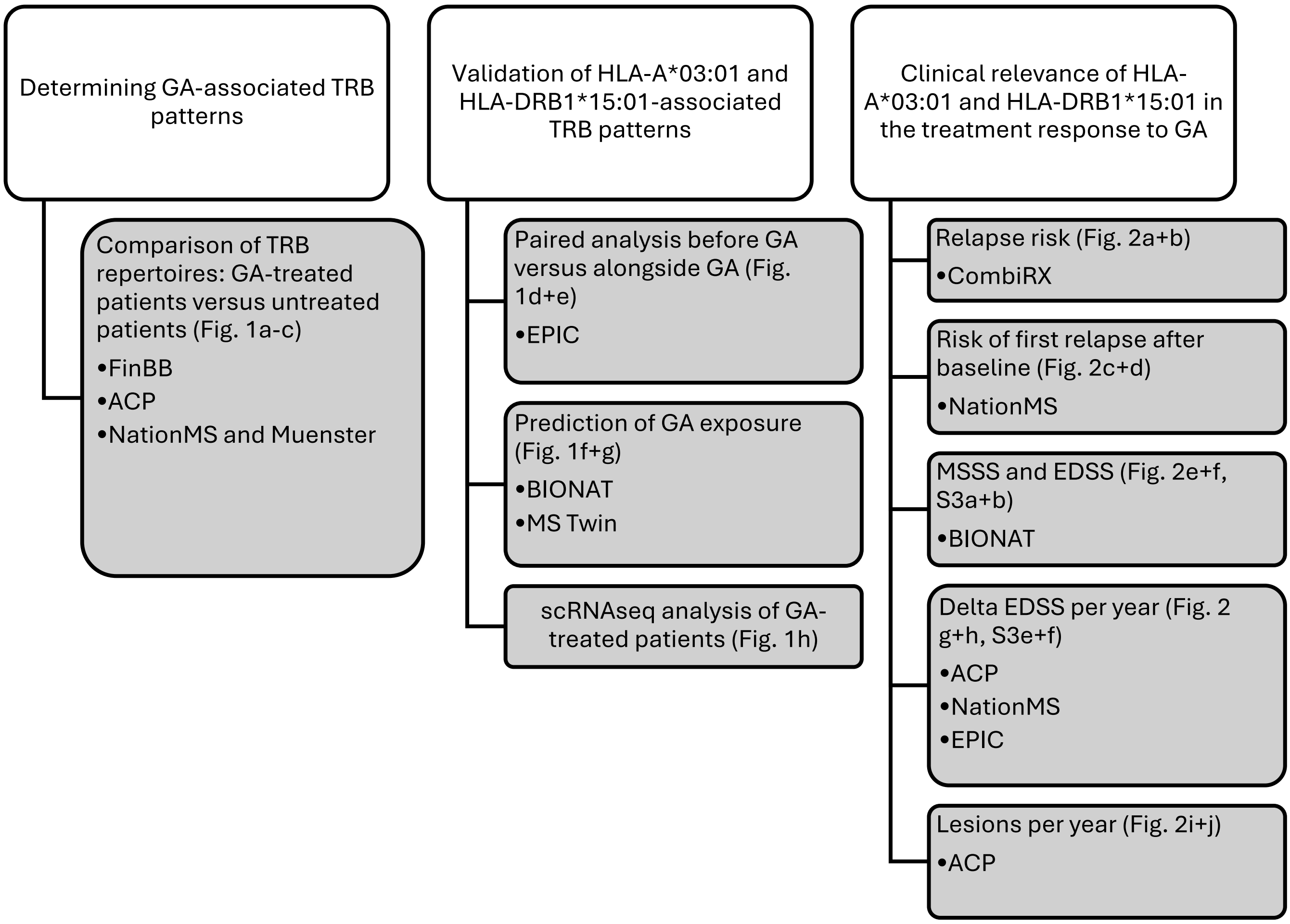
Determining GA-associated TRB patterns
Comparison of TRB repertoires: GA-treated patients versus untreated patients (Fig. 1a-c)
•FinBB
•ACP
•NationMS and Muenster
Validation of HLA-A*03:01 and HLA-DRB1*15:01-associated TRB patterns
Paired analysis before GA versus alongside GA (Fig. 1d+e)
•EPIC
Prediction of GA exposure (Fig. 1f+g)
•BIONAT
•MS Twin
scRNAseq analysis of GA-treated patients (Fig. 1h)
Clinical relevance of HLA-A*03:01 and HLA-DRB1*15:01 in the treatment response to GA
Relapse risk (Fig. 2a+b)
•CombiRX
Risk of first relapse after baseline (Fig. 2c+d)
•NationMS
MSSS and EDSS (Fig. 2e+f, S3a+b)
•BIONAT
Delta EDSS per year (Fig. 2 g+h, S3e+f)
•ACP
•NationMS
•EPIC
Lesions per year (Fig. 2i+j)
•ACP

Figure S6: Analytical sequence of the study

Schematic outline of the analytical sequence of the study: White squares indicate three analytical parts including discovery (left), validation (middle), and clinical evaluation (right) of GA-associated TRB sequences and their HLA restriction elements, grey squares indicate study cohorts and methodology utilised for the three analytical parts.
